# Abnormal DNA methylation within genes of the steroidogenesis pathway two years after paediatric critical illness and association with stunted growth in height further in time

**DOI:** 10.1186/s13148-023-01530-9

**Published:** 2023-07-19

**Authors:** Ilse Vanhorebeek, Grégoire Coppens, Fabian Güiza, Inge Derese, Pieter J. Wouters, Koen F. Joosten, Sascha C. Verbruggen, Greet Van den Berghe

**Affiliations:** 1 Clinical Division and Laboratory of Intensive Care Medicine, Department of Cellular and Molecular Medicine, Louvain, Herestraat 49, B-3000 Leuven, Belgium; 2 Division of Paediatric ICU, Department of Neonatal and Paediatric ICU, Erasmus Medical Centre, Sophia Children’s Hospital, Rotterdam, The Netherlands

**Keywords:** Critical illness, Paediatrics, Children, Steroidogenesis, Sex steroids, Aldosterone, Cortisol, DNA methylation, Growth, Height

## Abstract

**Background:**

Former critically ill children show an epigenetic age deceleration 2 years after paediatric intensive care unit (PICU) admission as compared with normally developing healthy children, with stunted growth in height 2 years further in time as physical correlate. This was particularly pronounced in children who were 6 years or older at the time of critical illness. As this age roughly corresponds to the onset of adrenarche and further pubertal development, a relation with altered activation of endocrine pathways is plausible. We hypothesised that children who have been admitted to the PICU, sex- and age-dependently show long-term abnormal DNA methylation within genes involved in steroid hormone synthesis or steroid sulphation/desulphation, possibly aggravated by in-PICU glucocorticoid treatment, which may contribute to stunted growth in height further in time after critical illness.

**Results:**

In this preplanned secondary analysis of the multicentre PEPaNIC-RCT and its follow-up, we compared the methylation status of genes involved in the biosynthesis of steroid hormones (aldosterone, cortisol and sex hormones) and steroid sulphation/desulphation in buccal mucosa DNA (Infinium HumanMethylation EPIC BeadChip) from former PICU patients at 2-year follow-up (*n* = 818) and healthy children with comparable sex and age (*n* = 392). Adjusting for technical variation and baseline risk factors and corrected for multiple testing (false discovery rate < 0.05), former PICU patients showed abnormal DNA methylation of 23 CpG sites (within *CYP11A1*, *POR*, *CYB5A*, *HSD17B1*, *HSD17B2*, *HSD17B3*, *HSD17B6*, *HSD17B10*, *HSD17B12*, *CYP19A1*, *CYP21A2*, and *CYP11B2*) and 4 DNA regions (within *HSD17B2*, *HSD17B8*, and *HSD17B10*) that were mostly hypomethylated. These abnormalities were partially sex- (1 CpG site) or age-dependent (7 CpG sites) and affected by glucocorticoid treatment (3 CpG sites). Finally, multivariable linear models identified robust associations of abnormal methylation of steroidogenic genes with shorter height further in time, at 4-year follow-up.

**Conclusions:**

Children who have been critically ill show abnormal methylation within steroidogenic genes 2 years after PICU admission, which explained part of the stunted growth in height at 4-year follow-up. The abnormalities in DNA methylation may point to a long-term disturbance in the balance between active sex steroids and mineralocorticoids/glucocorticoids after paediatric critical illness, which requires further investigation.

**Supplementary Information:**

The online version contains supplementary material available at 10.1186/s13148-023-01530-9.

## Background

Children who have been critically ill face substantial risk of long-term health problems and impairments in physical, neurocognitive and emotional/behavioural development, up to years after hospital discharge [1–8]. The mechanisms explaining adverse long-term outcomes after critical illness remain largely unclear, but epigenetic abnormalities induced by critical illness or associated treatments have been suggested as a plausible molecular basis [9]. Interestingly, estimation of “epigenetic” or biological age, with an epigenetic clock developed for children on buccal mucosa DNA [10], revealed an age deceleration in former critically ill children 2 years after PICU admission, as compared with normally developing healthy children, with stunted growth in height 2 years further in time as a physical correlate [11]. Vulnerability towards epigenetic age deceleration, interpreted as a developmental delay, was particularly observed from the age of 6 years onwards at the time of critical illness. This age window roughly corresponds with the time of adrenarche and further pubertal development.

Adrenarche is an early stage of sexual maturation, preceding puberty, in which the adrenal cortical zonation is completed with the establishment of the zona reticularis and in which adrenal steroid hormone production is consequently altered [12–15]. Whereas the zona glomerulosa is committed to mineralocorticoid (aldosterone) production and the zona fasciculata to glucocorticoid (cortisol) production, the appearance of the zona reticularis leads to an increase in the secretion of adrenal androgens. Gonadal steroid hormone production follows with pubertal development [16, 17]. The steroid hormones are produced from cholesterol via different branches of the steroidogenesis pathway and regulate a wide variety of developmental and physiological processes, including physical growth, from foetal life to adulthood [18, 19]. Hence, it is not surprising that a disturbed adrenarche and pubertal development may lead to health problems later in life, such as endocrine/metabolic abnormalities, cardiovascular disease and psychological problems, and may affect growth rate and final height [17, 20, 21].

As the age from which we observed particular vulnerability towards epigenetic age deceleration corresponded to the time of adrenarche and further pubertal development, a relation with altered activation of endocrine pathways at this time, which alters steroid production, is plausible. Another reason why disturbances in steroid production may be expected lies in the fact that children in the PICU are often treated with glucocorticoids [22]. Indeed, glucocorticoid treatment has been shown to suppress adrenal and gonadal steroidogenesis [23–26]. In this study, we therefore hypothesised that children who have been admitted to the PICU, age- and sex-dependently show long-term abnormal DNA methylation within genes involved in the biosynthesis or sulphation/desulphation of steroids, which may be affected by glucocorticoid treatment in the PICU. In addition, we hypothesised that abnormal methylation of the studied genes may contribute to stunted growth in height further in time after critical illness.

## Methods

### Study design and participants

**Fig. 1﻿:**
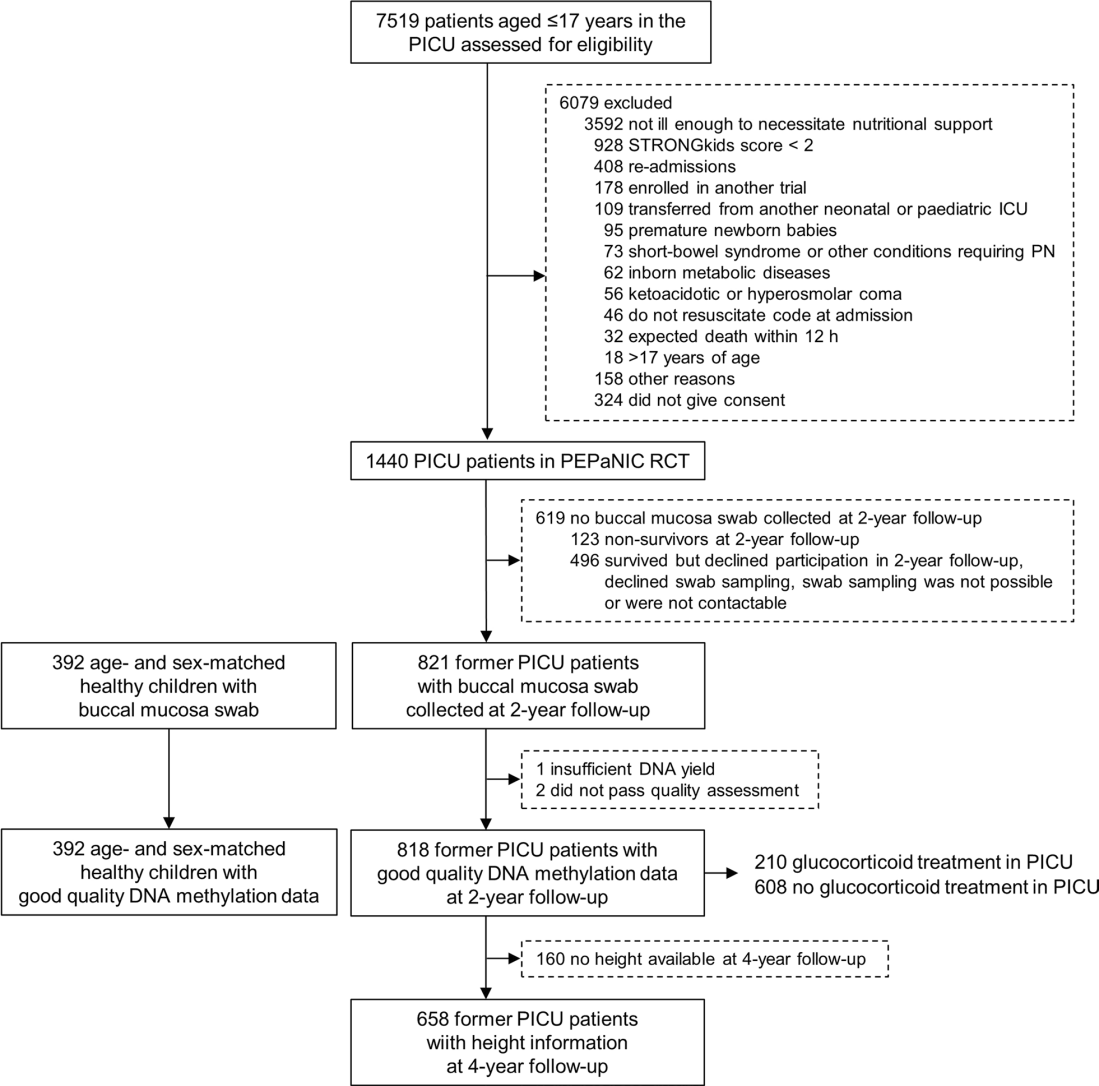
Diagram of study participants. PICU: paediatric intensive care unit, PEPaNIC: Paediatric Early versus Late Parenteral Nutrition in Intensive Care Unit, RCT: randomised controlled trial

This is a preplanned secondary analysis of the multicentre PEPaNIC randomised controlled trial and its 2- and 4-year follow-up studies (ClinicalTrials.gov, NCT01536275). The PEPaNIC-RCT enrolled 1440 critically ill children (age 0–17 years) between June 2012 and July 2015 in the PICUs of the University Hospitals Leuven (Belgium), Erasmus MC Sophia Children’s Hospital in Rotterdam (The Netherlands) and Stollery Children’s Hospital in Edmonton (Canada) to investigate the impact of a nutritional intervention on patient outcome [27, 28]. Patients were eligible for inclusion in this RCT if they had an expected PICU stay of at least 24 h, were at risk of malnutrition, and did not meet any of the exclusion criteria (Fig. 1). All surviving PEPaNIC patients were eligible for a pre-planned long-term follow-up 2 years (August 2014–January 2018) and 4 years (March 2016–November 2019) after PICU admission, assessing health status and physical and neurocognitive development [6, 7]. A control group of healthy children, comparable to the patients for sex and age, underwent identical longitudinal assessments. Apart from unrelated children, healthy siblings and patients’ relatives were included to control as much as possible for genetic, socioeconomic, and environmental background. Healthy children could only participate if they had not been previously admitted to a neonatal ICU or PICU, had not been admitted to hospital with need for an intravenous line for 7 days or more, and did not have a history of inborn chronic metabolic diseases requiring a specific diet (e.g. diabetes), or of conditions that require home parenteral nutrition (e.g. short-bowel syndrome). At the time of the 2-year follow-up, buccal mucosa swabs (Isohelix, Cell Projects, Harrietsham, Kent, England) were collected from the former PEPaNIC patients and the healthy children recruited in Leuven or Rotterdam. Swabs were collected following a standardised collection procedure and stored in a DNA stabilising solution (DSK kit, Isohelix) at −80 °C until further processing.

The institutional review boards at the participating sites approved the study (Ethische Commissie Onderzoek UZ Leuven/KU Leuven: ML8052; Medische Ethische Toetsingscommissie Erasmus MC: NL49708.078), which was performed in accordance with the 1964 Declaration of Helsinki and its amendments. Written informed consent was obtained from the parents or legal guardians, or from the children if 18 years or older.

### DNA extraction and DNA methylation data processing

As previously described [29], DNA was extracted from all available buccal mucosa swabs from patients and healthy children (DDK DNA isolation kit, Isohelix). DNA concentrations were quantified with the Qubit® 3.0 fluorometer (Thermo Fisher Scientific, Waltham, MA). Two-hundred ng DNA was subjected to bisulphite conversion with use of the EZ-96 DNA Methylation-Direct® Kit (Zymo Research, Irvine, CA). Bisulphite-converted DNA was profiled using the Infinium® HumanMethylation EPIC BeadChip (Illumina Inc., San Diego, CA), which interrogates 865,859 CpG sites. Data were processed using R statistical software version 4.2.2 using the LICMEpigenetics package (version 0.1.0) [30, 31]. This package contains R functions based on the Minfi pipeline [32–34] to exclude low-quality samples (not showing the typical bi-peak curve of the methylation value distribution in the low- and high-end range on the sample histograms) and low-quality probes (probes that did not exceed the background signal and probes spanning known single nucleotide polymorphisms), and to normalise the methylation data, adjust for batch effect and find differentially methylated positions and regions, as described below. Non-biological or technical variation due to experimental conditions was corrected for by including the first 30 principal components (PCs) of the control probes located on the Infinium® HumanMethylation EPIC BeadChip, excluding the negative control probes, as covariates in all multivariable linear regression models [29, 35].

### Selected genes

**Fig. 2﻿:**
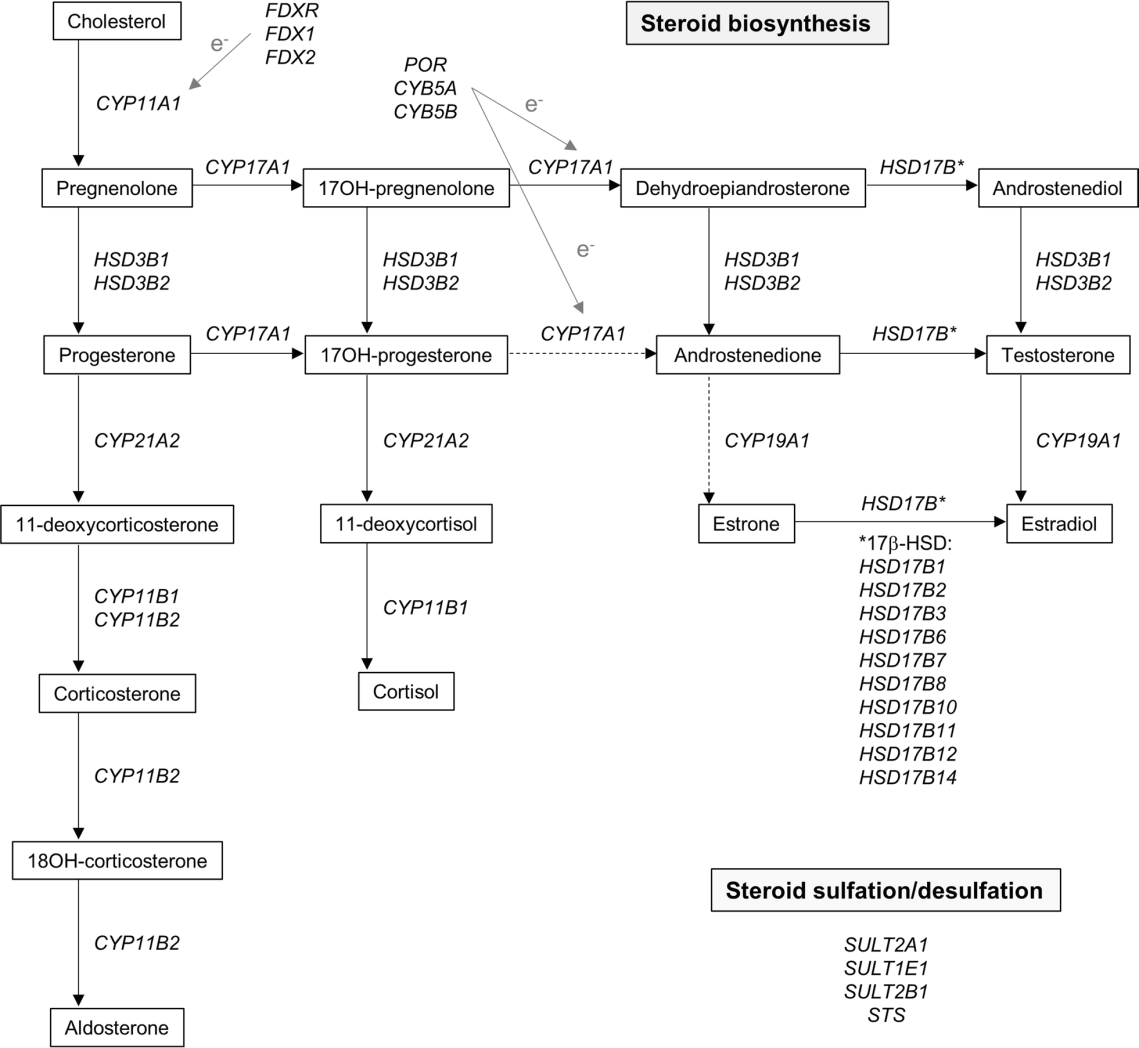
Schematic overview situating the studied genes within the different branches of the steroidogenesis pathway. *CYP11A1* encodes the first and rate-limiting enzyme in the steroidogenic pathway. *FDX1*, *FDX2* and *FDXR* encode ferredoxin (also called adrenodoxin) and ferredoxin reductase which are involved in electron transfer to CYP11A1. *HSD3B1* and *HSD3B2* encode the 3β-hydroxysteroid dehydrogenases that are involved in the synthesis of sex steroids, aldosterone and cortisol. *CYP17A1* encodes the enzyme catalysing the next step towards sex steroid production and *POR*, *CYB5A* and *CYB5B* encoding P450 oxidoreductase and cytochrome b5 are needed as mediators of electron transport to CYP17A1 for its 17,20-lyase activity. *HSD17B1*, *HSD17B2*, *HSD17B3*, *HSD17B6*, *HSD17B7*, *HSD17B8*, *HSD17B10*, *HSD17B11*, *HSD17B12* and *HSD17B14* encode several 17β-hydroxysteroid dehydrogenases and are a group of enzymes that mainly catalyse the interconversion of dehydroepiandrosterone <—> androstenediol, androstenedione <—> testosterone, and estrone <—> estradiol, with enzyme-specific substrate specificity/preferential direction of the reaction (see Additional File 1 Table A1). *CYP19A1* encodes aromatase which is responsible for the conversion of male to female sex hormones. *CYP21A2* encodes 21-hydroxylase and *CYP11B1* encodes 11β-hydroxylase that are required for the synthesis of both aldosterone and cortisol. *CYP11B2* encodes 11β-hydroxylase which is committed to the final steps of aldosterone synthesis. *SULT2A1*, *SULT2B1*, *SULT1E1* encode enzymes responsible for sulphation of steroids and *STS* encodes steroid sulphatase

For this study, we selected the CpG sites located in genes involved in steroid biosynthesis and steroid sulphation or desulphation [18, 19]. We analysed a total of 627 CpG sites in 28 genes. Figure 2 shows a schematic overview positioning the studied genes within the different branches of the steroidogenesis pathway. More information on the actions/enzyme activities of the corresponding proteins and on the CpG sites investigated within each gene is given in Additional file 1: Tables A1 and A2. The studied genes were *CYP11A1*, *FDX1*, *FDX2*, *FDXR*, *HSD3B1*, *HSD3B2*, *CYP17A1*, *POR*, *CYB5A*, *CYB5B*, *HSD17B1*, *HSD17B2*, *HSD17B3*, *HSD17B6*, *HSD17B7, HSD17B8*, *HSD17B10*, *HSD17B11*, *HSD17B12*, *HSD17B14*, *CYP19A1*, *CYP21A2*, *CYP11B1*, *CYP11B2*, *SULT2A1*, *SULT2B1*, *SULT1E1* and *STS*.

### Statistical analyses

Demographics and medical characteristics of the participants are presented as number (percentage) or median (interquartile range) and were compared between groups with Chi-square (Fisher exact) or Wilcoxon rank-sum tests, as appropriate, with use of JMP©Pro17.0.0 (SAS Institute, Inc, Cary, NC). Two-sided *P*-values < 0.05 were considered to indicate statistical significance for these comparisons. All other statistical analyses were performed in R version 4.2.2.

#### DNA methylation differences between former PICU patients and healthy children

We identified differences in DNA methylation between former PICU patients and healthy children via two approaches, focussing on methylation of individual CpG sites (identifying differentially methylated positions or DMPs) or of DNA regions (identifying groups of neighbouring CpG sites that are differentially methylated, called differentially methylated regions or DMRs). Such differences are to be considered the sum of differences evoked by the critical illness and intensive medical care and those that may have been present in former patients prior to PICU admission.

For the comparison of the degree of methylation of individual CpG sites between former PICU patients and healthy children (i.e. to identify DMPs), a multivariable linear regression model was built for each CpG site, with use of the limma framework [36]. The models were adjusted for baseline risk factors [age, treatment centre, sex, race, geographic origin, history of malignancy, predefined syndrome (Additional file 1: Methods A1)] and for technical variation (batch effect, vide supra [35]). Correction for multiple testing was done with a false discovery rate (FDR) < 0.05, as determined with the Benjamini–Hochberg procedure [37].

DMRs in former PICU patients as compared with the healthy children were identified with the DMRcate package, as previously described [29, 38]. This procedure involved calculation of a t-statistic with multivariable regression modelling comparing patients with the healthy children (adjusted for the above-mentioned baseline risk factors and for batch effect [35]), calculation of a weighted average for every CpG site (kernel estimate), comparison of the kernel estimates against a null comparison to assess statistical significance, and final determination of a differentially methylated region by grouping all significantly different kernel estimates not further than 1000 base pairs separated from one another. The two CpG locations within this group with the largest distance between each other determined the location and width of the differentially methylated region within the genome. A stepwise explanation of this method with an illustrative example is provided in Additional file 1: Methods A2.

#### Interaction with sex and age

For the differentially methylated positions in former PICU patients as compared with healthy children, we assessed whether there was an interaction with sex and/or age at exposure to critical illness and its treatments. We recently found that vulnerability to age deceleration of former PICU patients as compared with healthy children started from ± 6 years at exposure to critical illness and its treatments [11]. Therefore, we dichotomised age accordingly for the age-interaction analyses.

#### Role of glucocorticoid treatment during PICU stay

To investigate to what extent glucocorticoid treatment during PICU stay may have played a role in bringing about or aggravating any of the identified DMPs two years after PICU admission, we performed multivariable analyses with the limma framework, among former PICU patients, with the methylation status of each of the CpG sites identified above as the dependent variable, comparing glucocorticoid treatment versus no glucocorticoid treatment in the PICU. We adjusted for baseline risk factors and technical variation as described above, and for length of PICU stay, admission diagnosis, severity of illness [Paediatric Index of Mortality 3 (PIM3) score, Paediatric Logistic Organ Dysfunction (PeLOD) score], randomisation to 1 of 2 nutritional strategies, and risk of malnutrition [STRONGkids score].

#### Association with growth in height

The age deceleration that we had documented in former critically ill children 2 years after PICU admission, as compared with normally developing healthy children, had a physical correlate in the form of stunted growth in height 2 years further in time [11]. Therefore, we investigated whether methylation status in former PICU patients associated with height at 4-year follow-up. For each of the DMPs in former PICU patients as compared with healthy children, we thus built multivariable linear models for height at 4-year follow-up, adjusting for baseline risk factors [age, treatment centre, sex, race, geographic origin, history of malignancy, predefined syndrome (Additional file 1: Methods A1), type and severity of illness [Paediatric Index of Mortality 3 (PIM3) score, Paediatric Logistic Organ Dysfunction (PeLOD) score], randomisation to 1 of 2 nutritional strategies, and risk of malnutrition [Screening Tool for Risk on Nutritional Status and Growth (STRONGkids) score])] and for technical variation [35]. Robustness of the linear models was assessed via tenfold cross-validation with computation of the P-values of the 10 test folds using Fisher’s method [39], and repetition of this process in 100 iterations. The percentage of iterations with a significant *P*-value across the 10-folds of the cross-validation (α < 0.05) was calculated. An association was deemed robust if present in at least 50 of the iterations [40]. With use of the mean coefficient from the multivariable models, we further assessed whether the observed abnormal methylation in former PICU patients was associated with either a beneficial or harmful effect on height at 4-year follow-up.

## Results

Buccal mucosa swabs were available for 392 healthy children and for 821 patients at 2-year follow-up (Fig. 1). DNA yield was insufficient for 1 patient and 2 patients showed deviation from the typical bi-modal curve of the methylation value distribution, leaving 818 patients for the DNA methylation analyses [29], of whom 210 had received glucocorticoid treatment during PICU stay, and with height at further 4-year follow-up available for 658 patients. Participants’ demographics and medical characteristics of the former PICU patients are shown in Table 1.

**Table 1:** Participants’ demographics and medical characteristics

Demographics and medical characteristics	DNA methylation study at 2-year follow-up						PICU patients with height at 4-year follow-up N = 658
	Healthy children N = 392	PICU patients N = 818	*P*-value	PICU patients, no GC N = 608	PICU patients, GC N = 210	*P*-value	
Demographics							
Age at 2-year follow-up (years)—median (IQR)	3.8 (2.6–8.2)	3.4 (2.6–7.9)	0.96	3.2 (2.6–7.9)	3.6 (2.6–8.0)	0.31	3.3 (2.6–7.5)
Sex							
Male—no (%)	212 (54.1)	475 (58.1)	0.19	212 (54.1)	475 (58.1)	0.10	385 (58.5)
Female—no (%)	180 (45.9)	343 (41.9)		180 (45.9)	343 (41.9)		273 (41.5)
Known non-Caucasian race^a^—no (%)	32 (8.2)	66 (8.1)	0.95	42 (6.9)	24 (11.4)	0.045	52 (7.9)
Known non-European origin^a^—no (%)	51 (13.0)	144 (17.6)	0.038	96 (15.8)	48 (22.9)	0.023	109 (16.6)
Medical characteristics							
STRONGkids risk level^b^			–			0.30	
Medium—no (%)	NA	736 (90.0)		551 (90.6)	185 (88.1)		588 (89.4)
High—no (%)	NA	82 (10.0)		57 (9.4)	25 (11.9)		70 (10.6)
PeLOD score, first 24 h in PICU^c^—median (IQR)	NA	22 (12–32)	–	22 (12–32)	21 (11–31)	0.012	21 (12–32)
PIM3 score^d^—median (IQR)	NA	-3.8 (-4.4 to -2.7))	–	-3.9 (-4.4 to -2.9)	-3.0 (-4.3 to -0.9)	< 0.0001	-3.7 (-4.4 to -2.7)
PIM3 probability of death^e^ (%)—median (IQR)	NA	2.3 (1.2–6.5)	–	2.0 (1.2–5.3)	4.6 (1.4–10.4)	< 0.0001	2.3 (1.2–6.5)
Diagnostic category			–			< 0.0001	
Cardiac surgery—no (%)	NA	364 (44.5)		308 (50.7)	56 (26.7)		
Elective other surgery—no (%)	NA	116 (14.2)		85 (14.0)	31 (14.8)		
Urgent other surgery—no (%)	NA	142 (17.4)		96 (15.8)	46 (21.9)		
Medical diagnosis—no (%)	NA	196 (24.0)		119 (19.6)	77 (36.7)		
Malignancy—no (%)	0 (0.0)	39 (4.8)	< 0.0001	18 (3.0)	21 (10.0)	0.0001	36 (5.5)
Syndrome^e^—no (%)	4 (1.0)	168 (20.5)	< 0.0001	118 (19.4)	50 (23.8)	0.17	120 (18.2)

*IQR* interquartile range; *GC* glucocorticoid treatment during PICU stay; *NA* not applicable; *no* number; *PeLOD* Paediatric Logistic Organ Dysfunction score; *PICU* paediatric intensive care unit; *PIM3* Paediatric Index of Mortality 3 score; *PN* parenteral nutrition; *SD* standard deviation; *STRONGkids* Screening Tool for Risk on Nutritional Status and Growth for kids

^a^Participants were classified according to race and geographical origin by the investigators

^b^STRONGkids scores range from 0 to 5, with a score of 0 indicating a low risk of malnutrition, a score of 1 to 3 indicating medium risk, and a score of 4 to 5 indicating high risk

^c^PeLOD scores range from 0 to 71, with higher scores indicating more severe illness

^d^Higher PIM3 scores indicate a higher risk of mortality

^e^A pre-randomisation syndrome or illness a priori defined as affecting or possibly affecting development (Additional file 1: Methods A1)

### Differential DNA methylation within steroidogenic genes in former PICU patients versus healthy children

**Fig. 3:**
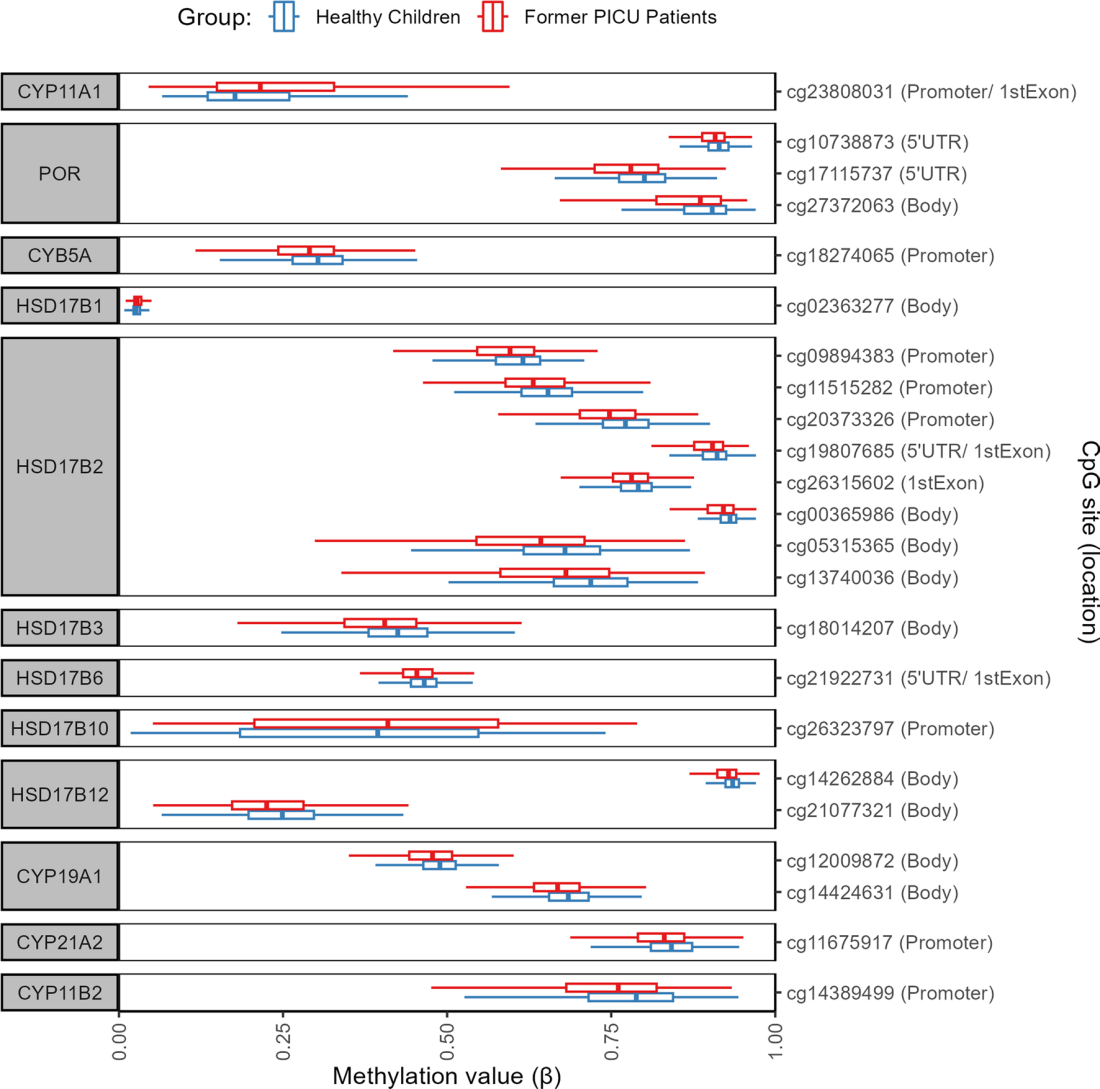
Methylation status of differentially methylated positions in former PICU patients as compared with healthy children. The boxplots show a univariate presentation of the methylation status (β-value) of the CpG sites that were differentially methylated between former PICU patients (red) and matched healthy children (blue). The CpG sites are grouped per gene and positioned based on their location within the gene. The central lines of the boxplots depict the medians, the boxes the interquartile ranges, and the whiskers are drawn to the furthest point within 1.5 times the interquartile range from the box. PICU: paediatric intensive care unit, UTR: untranslated region

Corrected for multiple testing, we observed differential methylation in former PICU patients as compared with healthy children at the level of 23 CpG sites (all *P* ≤ 0.049). Table 2 summarises the location of those 23 CpG sites within the genome, the direction of the difference, i.e. whether they are hypomethylated or hypermethylated in former PICU patients as compared with healthy children, and the effect size. The absolute mean differences in DNA methylation beta-values for the DMPs were 2.3% (SD 1.2%), ranging up to 5.2%, with mostly hypomethylation in former PICU patients (20/23 (87.0%), Table 2, Fig. 3). The 23 CpG sites differentially methylated in former PICU patients as compared with healthy children were located in 12 of the 28 selected genes (42.9%), more specifically *CYP11A1* (1/37 CpG sites, 2.7%), *POR* (3/69 CpG sites, 4.4%), *CYB5A* (1/28 CpG sites, 3.6%), *HSD17B1* (1/15 CpG sites, 6.7%), *HSD17B2* (8/32 CpG sites, 25.0%), *HSD17B3* (1/19 CpG sites, 5.3%), *HSD17B6* (1/10 CpG sites, 10.0%), *HSD17B10* (1/15 CpG sites, 6.7%), *HSD17B12* (2/47 CpG sites, 4.3%), *CYP19A1* (2/55 CpG sites, 3.6%), *CYP21A2* (1/13 CpG sites, 7.7%), and *CYP11B2* (1/11 CpG sites, 9.1%) (Additional file 1: Table A2).

**Table 2:** Abnormal DNA methylation within the steroidogenesis pathway

Gene	CpG site	Gene section^a,b^	Methylation status	Former PICU patients vs healthy children					Former PICU patients: GC vs No GC treatment *P*-value^e^
				Log fold change [confidence interval]^c^	Absolute mean difference^d^	*P*-value^e^			
						Patients vs healthy children ^f^	Interaction with sex	Interaction with age	
CYP11A1	cg23808031	Promoter/1stExon	Hypermethylated	0.195 [0.090 to 0.301]	0.041	**0.016**	0.51	0.82	**0.0090**
POR	cg10738873	5'UTR	Hypomethylated	−0.116 [−0.175 to −0.058]	0.011	**0.0092**	0.69	0.26	0.22
	cg17115737	5'UTR	Hypomethylated	−0.099 [−0.159 to −0.039]	0.026	**0.036**	0.49	0.84	0.84
	cg27372063	Body	Hypomethylated	−0.182 [−0.286 to −0.078]	0.026	**0.026**	0.29	0.24	0.068
CYB5A	cg18274065	Promoter	Hypomethylated	−0.133 [−0.196 to −0.071]	0.019	**0.0067**	0.32	0.79	0.18
HSD17B1	cg02363277	Body	Hypermethylated	0.039 [0.015 to 0.062]	0.002	**0.044**	0.80	0.61	0.34
HSD17B2	cg09894383	Promoter	Hypomethylated	−0.080 [−0.126 to −0.035]	0.023	*0.026*	0.30	**0.012**	0.98
	cg11515282	Promoter	Hypomethylated	−0.100 [−0.148 to −0.052]	0.023	**0.0067**	0.76	**0.029**	0.52
	cg20373326	Promoter	Hypomethylated	−0.135 [−0.190 to −0.080]	0.027	**0.0009**	0.84	0.36	0.34
	cg19807685	5'UTR/1stExon	Hypomethylated	−0.124 [−0.182 to −0.065]	0.011	**0.0067**	0.47	0.18	0.84
	cg26315602	1stExon	Hypomethylated	−0.073 [−0.113 to −0.034]	0.010	**0.016**	0.76	0.93	0.66
	cg00365986	Body	Hypomethylated	−0.134 [−0.212 to −0.057]	0.016	**0.026**	0.99	0.25	**0.045**
	cg05315365	Body	Hypomethylated	−0.170 [−0.259 to −0.082]	0.046	**0.013**	0.55	**0.017**	0.23
	cg13740036	Body	Hypomethylated	−0.192 [−0.287 to −0.098]	0.052	**0.0073**	0.46	**0.045**	0.68
HSD17B3	cg18014207	Body	Hypomethylated	−0.131 [−0.204 to −0.058]	0.031	**0.025**	0.89	0.13	0.056
HSD17B6	cg21922731	5'UTR/1stExon	Hypomethylated	−0.044 [−0.069 to −0.018]	0.010	**0.026**	0.44	0.87	0.68
HSD17B10	cg26323797	Promoter	Hypermethylated	0.146 [0.062 to 0.231]	0.024	**0.026**	0.47	0.32	0.18
HSD17B12	cg14262884	Body	Hypomethylated	−0.081 [−0.131 to −0.030]	0.010	**0.049**	0.38	**0.048**	0.66
	cg21077321	Body	Hypomethylated	−0.133 [−0.212 to −0.055]	0.025	**0.031**	0.86	0.081	0.68
CYP19A1	cg12009872	Body	Hypomethylated	−0.057 [−0.092 to −0.021]	0.015	**0.046**	0.93	**0.012**	0.67
	cg14424631	Body	Hypomethylated	−0.093 [−0.138 to −0.048]	0.020	**0.0072**	0.85	**0.035**	**0.0027**
CYP21A2	cg11675917	Promoter	Hypomethylated	−0.129 [−0.199 to −0.060]	0.019	**0.016**	**0.043**	0.24	0.44
CYP11B2	cg14389499	Promoter	Hypomethylated	−0.100 [−0.159 to −0.041]	0.031	**0.031**	0.88	0.41	0.14

*GC* glucocorticoid; *PICU* paediatric intensive care unit, *UTR* untranslated region

^a^A CpG site can be located within multiple splice variants and thus can be situated within multiple gene sections

^b^Promoter is defined as 0 to 1500 base pairs upstream of the transcription start site

^c^Log fold change in M-values between former PICU patients and healthy controls adjusted for risk factors with 95% confidence interval

^d^The absolute mean difference is the (unadjusted) absolute difference between the mean beta-value of a given CpG within the Former PICU patients and the mean beta-value of a given CpG within the healthy control children

^e^P-values from multivariable models, adjusted for baseline risk factors and technical variation. All P-values come from separate models. Numbers in bold indicate a significant P-value

^f^Adjusted for multiple testing using a false discovery rate < 0.05

**Fig. 4:**
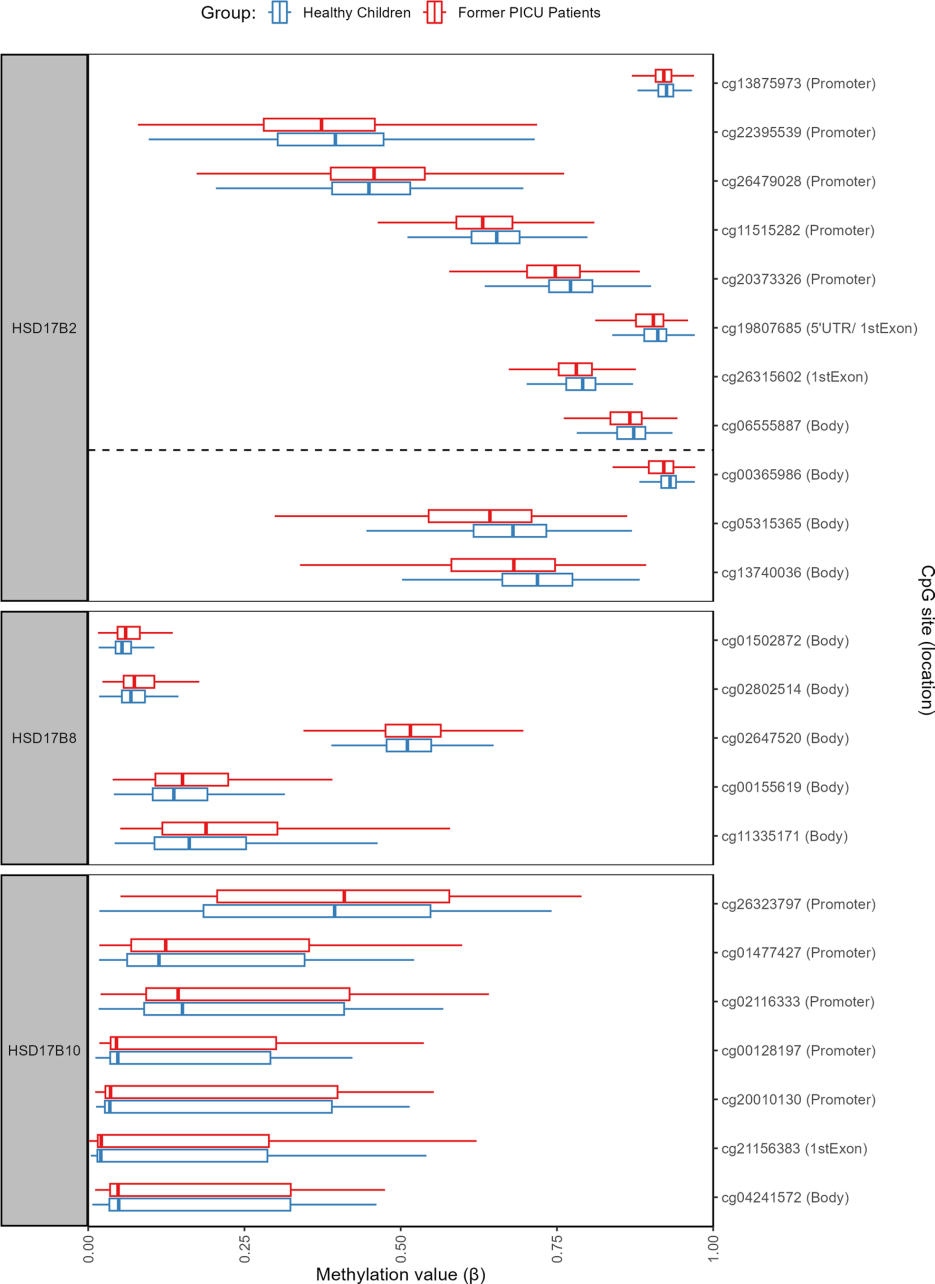
Methylation status of CpG sites within regions differentially methylated between former PICU patients and healthy children. The boxplots show a univariate presentation of the methylation status (β-value) of the CpG sites located within the regions of the *HSD17B2*, *HSD17B8* and *HSD17B10* genes identified as differentially methylated between former PICU patients (red) and matched healthy children (blue). The CpG sites are grouped per gene and positioned based on their location within the gene. The central lines of the boxplots depict the medians, the boxes the interquartile ranges, and the whiskers are drawn to the furthest point within 1.5 times the interquartile range from the box. PICU: paediatric intensive care unit

In addition, four DMRs were identified, two located within the *HSD17B2* gene, one in the *HSD17B8* gene and one in the *HSD17B10* gene (Fig. 4, Additional file 1: Table A3). Both DMRs within the *HSD17B2* gene were hypomethylated in former PICU patients as compared with healthy children. The first spanned a width of 1167 base pairs and contained 8 CpG sites; the second spanned a width of 40 base pairs and contained 3 CpG sites. All DMPs identified within the *HSD17B2* gene, except cg09894383, were part of a DMR. The other DMRs were hypermethylated in former PICU patients as compared with healthy children. The DMR within the *HSD17B8* gene spanned a width of 215 base pairs and contained 5 CpG sites, the DMR within the *HSD17B10* gene spanned a width of 250 base pairs and contained 7 CpG sites (including the DMP identified above).

Combining the identified DMPs and DMRs, differential methylation in former PICU patients as compared with healthy children affected 13 of the 28 selected genes (46.4%).

### Interaction with sex and age “at exposure”

**Fig. 5:**
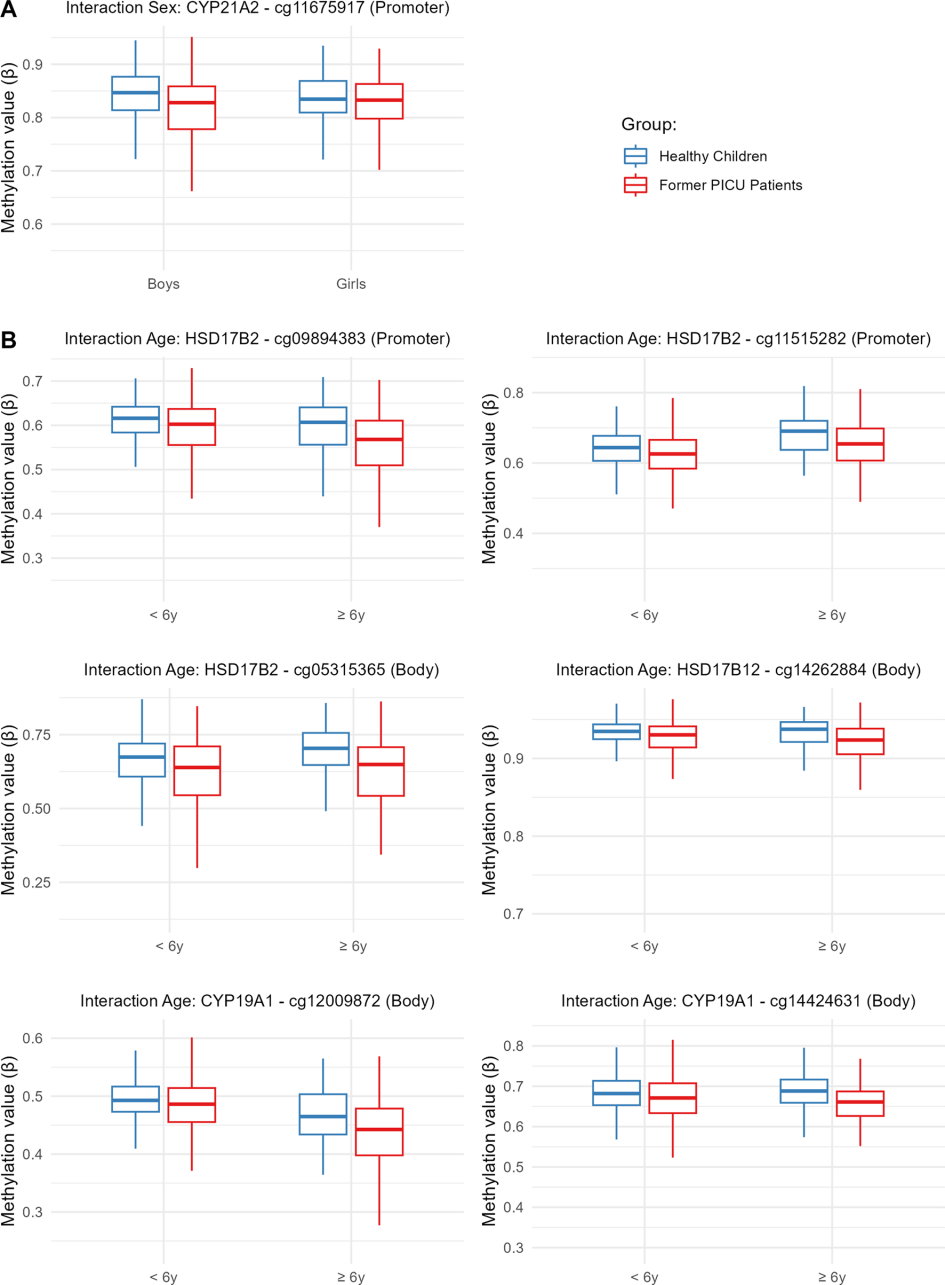
Interaction with sex or age of abnormal DNA methylation in former PICU patients vs healthy children. The boxplots show a univariate presentation of the methylation status (β-value) of the CpG sites for which, adjusting for baseline risk factors and technical variation, a significant interaction was found between differential methylation in former PICU patients (red) vs matched healthy children (blue) and sex (panel A) or “age at exposure” (panel B). The central lines of the boxplots depict the medians, the boxes the interquartile ranges, and the whiskers are drawn to the furthest point within 1.5 times the interquartile range from the box. PICU: paediatric intensive care unit

Only 1 of the identified differences in DNA methylation between former PICU patients and matched healthy children was sex-dependent, namely that of cg11675917 within the promoter of *CYP21A2* (*P* = 0.043, Table 2, Additional file 1: Table A4). Hypomethylation of this CpG site in former PICU patients was more pronounced in boys than in girls (Fig. 5, panel A).

Age “at exposure” affected the methylation status of 7 of the differentially methylated CpG sites (Table 2, Additional file 1: Table A5). These CpG sites were located in the promoter region (cg09894383, *P* = 0.012 and cg11515282, *P* = 0.029) or gene body (cg05315365, *P* = 0.017 and cg13740036, *P* = 0.045) of *HSD17B2* and in the gene bodies of *HSD17B12* (cg14262884, *P* = 0.048) and *CYP19A1* (cg12009872, *P* = 0.012 and cg14424631, *P* = 0.035). The degree of hypomethylation of these CpG sites in former PICU patients as compared with healthy children was more pronounced in children of 6 years or older at exposure to critical illness and its treatments than in younger children (Fig. 5, panel B).

### Role of glucocorticoid treatment in the PICU

**Fig. 6:**
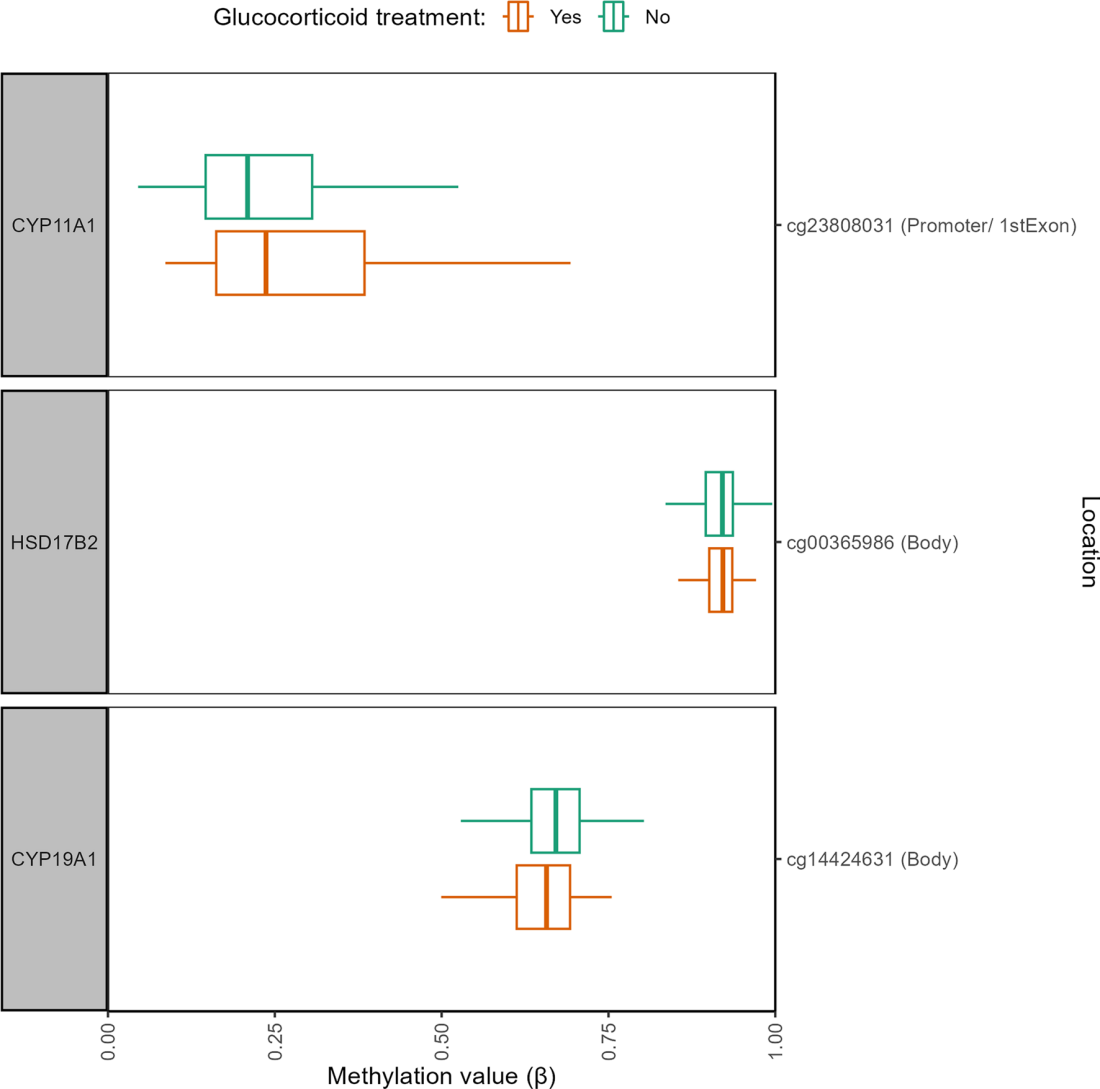
Differentially methylated CpG sites between former PICU patients who received glucocorticoids during their stay in the PICU versus those who did not. The boxplots show a univariate presentation of the methylation status (β-value) of the CpG sites for which, adjusting for baseline risk factors and technical variation, a significant difference was found between methylation status in former PICU patients who received glucocorticoid treatment in the PICU (orange) vs those who did not (green). The central lines of the boxplots depict the medians, the boxes the interquartile ranges, and the whiskers are drawn to the furthest point within 1.5 times the interquartile range from the box. PICU: paediatric intensive care unit

Three of the identified DMPs were affected by glucocorticoid treatment given during PICU stay (Table 2, Fig. 6, Additional file 1: Table A6). Abnormal methylation of cg23808031 in *CYP11A1* (*P* = 0.0090) and cg14424631 in *CYP19A1* (*P* = 0.0027) was aggravated, whereas that of cg00365986 in *HSD17B2* (*P* = 0.045) was attenuated by such glucocorticoid treatment. The absolute differences in DNA methylation beta-values for these CpG sites between former patients who had received glucocorticoid treatment and those who had not were 2.4% (SD 2.1%), ranging up to 4.5%.

### Association of abnormal DNA methylation within steroidogenic genes with growth in height of former PICU patients

Among the participants with DNA methylation data at 2-year follow-up, height at 4-year follow-up of former PICU patients (*n* = 658) was 112 (104–135) cm as compared with 114 (107–137) cm for healthy children (*n* = 346, *P* = 0.0024). The statistical association of DNA methylation differences between former PICU patients and healthy children with height at 4-year follow-up is shown in Table 3. For all 23 CpG sites, we found very robust associations of the degree of abnormal methylation within former PICU patients with a shorter height at 4-year follow-up, being significant in 100% of the iterations (75th percentile of the Fisher *P*-values across the 10 test folds obtained for the 100 iterations ≤ 0.016).

**Table 3:** Association with stunted growth in height of long-term abnormal DNA methylation within steroidogenic genes in former PICU patients

Gene	CpG site	Gene section ^a,b^	Percentage of significant iterations ^c^	Median (IQR) *P*-value ^d^	Direction of association ^e^
CYP11A1	cg23808031	Promoter/ 1stExon	100	2.8E-26 (1.8E-26–3.7E-26)	Harm
POR	cg10738873	5'UTR	100	2.2E-11 (1.7E-11–2.9E-11)	Harm
	cg17115737	5'UTR	100	6.3E-13 (5.0E-13–8.1E-13)	Harm
	cg27372063	Body	100	1.2E-24 (8.4E-25–1.5E-24)	Harm
CYB5A	cg18274065	Promoter	100	4.6E-29 (3.5E-29–6.4E-29)	Harm
HSD17B1	cg02363277	Body	100	1.7E-10 (1.4E-10–2.0E-10)	Harm
HSD17B2	cg09894383	Promoter	100	8.6E-40 (5.2E-40–1.2E-39)	Harm
	cg11515282	Promoter	100	8.3E-87 (4.7E-87–1.2E-86)	Harm
	cg20373326	Promoter	100	1.9E-75 (1.2E-75–3.0E-75)	Harm
	cg19807685	5'UTR/ 1stExon	100	1.5E-37 (1.0E-37–2.0E-37)	Harm
	cg26315602	1stExon	100	8.1E-28 (5.6E-28–1.3E-27)	Harm
	cg00365986	Body	100	6.4E-32 (4.3E-32–9.6E-32)	Harm
	cg05315365	Body	100	2.6E-80 (1.6E-80–5.0E-80)	Harm
	cg13740036	Body	100	2.5E-80 (1.6E-80–4.5E-80)	Harm
HSD17B3	cg18014207	Body	100	1.3E-25 (9.3E-26–1.7E-25)	Harm
HSD17B6	cg21922731	5'UTR/ 1stExon	100	8.7E-07 (6.6E-07–1.0E-06)	Harm
HSD17B10	cg26323797	Promoter	100	0.014 (0.013–0.016)	Harm
HSD17B12	cg14262884	Body	100	3.0E-20 (2.1E-20–4.2E-20)	Harm
	cg21077321	Body	100	7.3E-27 (5.6E-27–1.0E-26)	Harm
CYP19A1	cg12009872	Body	100	4.5E-14 (3.3E-14–6.1E-14)	Harm
	cg14424631	Body	100	8.9E-07 (7.2E-07–1.0E-06)	Harm
CYP21A2	cg11675917	Promoter	100	5.9E-09 (4.9E-09–6.8E-09)	Harm
CYP11B2	cg14389499	Promoter	100	4.7E-10 (3.8E-10–6.0E-10)	Harm

This table summarises the results of the multivariable linear regression analyses assessing associations between abnormal DNA methylation within genes involved in steroidogenesis 2 years after critical illness and height at 4-year follow-up, adjusted for baseline risk factors and technical variation

*UTR* untranslated region

^a^A CpG site can be located within multiple splice variants and thus can be situated within multiple gene sections

^b^Promoter is defined as 0 to 1500 base pairs upstream of the transcription start site

^c^Percentage of the 100 iterations for which the Fisher P-values across the 10 test folds is significant (*P* < 0.05)

^d^Median and interquartile range of the Fisher P-values across the 10 test folds obtained for the 100 iterations

^e^The label “Harm” for robust associations (significant in at least 50% of the iterations) indicates that the observed abnormal DNA methylation correlates with a shorter height (based on the mean coefficient from the multivariable models)

## Discussion

As compared with healthy children, former PICU patients assessed 2 years after critical illness showed abnormal methylation of buccal mucosa DNA at the level of several genes within the steroid biosynthesis pathway. The abnormal DNA methylation in former PICU patients was mostly hypomethylation, was only partially sex- or age-dependent and appeared in part aggravated by glucocorticoid treatment in the PICU. All DNA methylation abnormalities robustly associated with stunted growth in height 2 years further in time.

This study documented altered methylation in buccal mucosa DNA of former PICU patients 2 years after the acute critical illness within several genes involved in the biosynthesis of steroid hormones, but not in their sulphation or desulphation. Of the genes required for synthesis of all 3 studied types of steroid hormones, only DNA methylation of *CYP11A1* was affected. The remaining abnormal DNA methylation affected genes involved in specific branches of the steroidogenesis pathway, more specifically *CYP11B2* and *CYP21A2* committed to mineralocorticoid and/or glucocorticoid synthesis, and several genes committed to the synthesis of sex steroids. The latter included *POR* and *CYB5A* (needed for CYP17A1’s 17,20-lyase activity), the aromatase-encoding *CYP19A1*, and several genes encoding 17β-hydroxysteroid dehydrogenases which type-dependently convert inactive/weakly active sex steroids to active sex steroid hormones or vice versa. A widely used rule of thumb in literature is that promoter/1^st^ exon methylation inversely correlates with gene expression (although not always true), whereas a positive correlation between methylation of the gene body and gene expression has been suggested (although less uniformly documented) [41–43]. If the observed altered DNA methylation affects steroidogenic gene expression and corresponding enzyme activities, this could disturb the balance in production of the different steroid hormones. Unfortunately, we did not have the material to associate DNA methylation abnormalities with gene expression. However, other studies have documented correlations between methylation status and gene expression of *CYP11A1*, *CYP11B2*, *CYP19A1*, *HSD17B1* and *HSD17B2* [44–55]. We “speculate” on potential effects on gene expression of the observed differences in DNA methylation between former PICU patients and healthy children in Figure A1 (Additional file 1).

The abnormal DNA methylation of steroidogenic genes was sex-independent, except for hypomethylation of a CpG site within the *CYP21A2* promoter that was observed particularly in boys. Age dependency of vulnerability towards abnormal DNA methylation was somewhat greater, with a more pronounced hypomethylation of several CpG sites within *HSD17B2*, *HSD17B12* and *CYP19A1* in children who at the time of PICU admission had already reached or passed the age of adrenarche as compared with younger children. This corresponds with our earlier finding of a higher vulnerability to epigenetic age deceleration of former PICU patients from the age of adrenarche onwards and into puberty [11].

Glucocorticoid treatment given in the PICU was found to independently associate with abnormal methylation of 3 CpG sites. For hypermethylation within the promoter/first exon of *CYP11A1* and hypomethylation within the gene body of *CYP19A1* the association pointed to more abnormal methylation with exogenous glucocorticoids. In both cases, the more abnormal methylation likely coincides with downregulated gene expression [41–43], as several animal studies showed reduced adrenal and/or gonadal *CYP11A1* and *CYP19A1* expression with increased (exogenous) glucocorticoid exposure [56–63]. In contrast, hypomethylation of one CpG site within *HSD17B2* may have been somewhat attenuated by glucocorticoid treatment, the impact of which remains uncertain in the absence of literature data on gene expression to compare with and in view of the redundant activity of several 17β-hydroxysteroid dehydrogenases. Nevertheless, the possible aggravation of the abnormal methylation within the two other genes argues for caution, as it may question the safety of a liberal glucocorticoid use in the PICU in the absence of strong underlying evidence of benefit.

Interestingly, we observed strong associations of the abnormal DNA methylation within steroidogenic genes with the stunted growth in height of former PICU patients as compared with healthy children. Although, as mentioned earlier, we were unable to study the impact on corresponding gene expression, the strong associations with impaired growth in height, in combination with published evidence, may support the idea of an imbalance between production of active sex steroids versus aldosterone or cortisol years after paediatric critical illness. Indeed, steroid hormones have an important impact on longitudinal bone growth [64]. In that regard, an appropriate estradiol level is essential already in early childhood for a normal prepubertal growth rate and is needed for the pubertal growth spurt in both boys and girls [65–67]. Estrogens, rather than androgens, appear essential for harmonic skeletal growth in both sexes [64, 68, 69]. Thus, it is plausible that disturbed sex hormone production/function, possibly related to abnormal DNA methylation of steroidogenic genes, contributes to stunted growth in height years after paediatric critical illness. Also, excessive exposure to (endogenous or exogenous) glucocorticoids in childhood has shown to impair linear growth [70]. Critically ill children show elevated endogenous cortisol levels upon PICU admission and are often treated with glucocorticoids during PICU stay [22]. Long-term abnormal cortisol production may be suggested by the here observed abnormal DNA methylation within *CYP21A2*.

A strength of this study is the multicentre, prospective design with predefined long-term assessments of large cohorts of former PICU patients in parallel with matched healthy children. Another strength is the used methodology which reduced the odds of findings by chance and impact of outliers. Indeed, we applied an FDR of < 0.05 for the identification of differential methylation between groups and performed a tenfold cross validation over 100 iterations in the analyses assessing association with stunted growth in height. Our study also has limitations to highlight. First, due to obvious practical and ethical limitations, we used buccal mucosa to extract DNA from, which may not allow extrapolation to other tissues of interest, i.e. the adrenal gland or gonads where steroid hormones are mainly produced. Second, as mentioned, we were unable to study gene expression. Hence, it remains unclear whether the identified abnormal DNA methylation has an impact on gene transcription and thus our interpretation in this regard remains speculative. Also, information about the impact of abnormal DNA methylation of steroidogenic genes on hormone levels is lacking, as we did not collect blood samples from these young children because of ethical reasons. Nevertheless, the strong associations between the abnormal DNA methylation and the stunted growth in height years later supports functional relevance. Finally, glucocorticoid treatment during PICU stay had not been randomised. Although we extensively adjusted for risk factors which also included type, severity, and duration of illness, we cannot exclude that there may be some residual unmeasured confounding.

## Conclusions

We observed abnormal methylation of several CpG sites within genes of the steroid biosynthesis pathway in buccal mucosa DNA collected 2 years after critical illness in children, which was largely independent of sex and age, and which was partly attributable to glucocorticoid treatment in the PICU. The abnormalities in DNA methylation status may point to a long-term imbalance between active sex steroids and mineralocorticoids/glucocorticoids after paediatric critical illness, a possibility that requires further investigation, and explained part of the stunted growth in height further in time.

## Supplementary Information

**Additional file 1**: Compiled file with all Additional information: Additional Methods describing the definition of “Syndrome” and a stepwise explanation of the DMRcate method for the identification of differentially methylated DNA regions; Additional Tables listing the genes and number of CpG sites investigated for differential methylation between former PICU patients and healthy children, the differentially methylated regions in former PICU patients versus healthy children, the interaction of differential methylation in former PICU patients versus healthy children with sex and age at exposure, and the analyses of differential methylation between former PICU patients who received glucocorticoids during their stay in the PICU versus those who did not; and Additional Figure summarising a speculative interpretation of potential impact of abnormal DNA methylation within steroidogenic genes on corresponding gene expression.

## Data Availability

Data sharing is offered under the format of collaborative projects. Proposals can be directed to the corresponding author.

## Abbreviations

DMP: Differentially methylated position
DMR: Differentially methylated region
ICU: Intensive care unit
PeLOD: Paediatric Logistic Organ Dysfunction score
PEPaNIC: Paediatric Early versus Late Parenteral Nutrition in Intensive Care Unit
PICU: Paediatric intensive care unit
PIM3: Paediatric Index of Mortality 3 score
RCT: Randomised controlled trial
STRONGkids: Screening Tool for Risk on Nutritional Status and Growth for kids
