# Supplementary material for: Abnormal DNA methylation within genes of the steroidogenesis pathway two years after paediatric critical illness and association with stunted growth in height further in time
Source: Clin Epigenetics. 2023 Jul 19;15:116. doi: 10.1186/s13148-023-01530-9 (PMC10354984; doi:10.1186/s13148-023-01530-9)
Supplement: Supplementary file 1 — Additional file 1: Compiled file with all Additional information: Additional Methods describing the definition of “Syndrome” and a stepwise explanation of the DMRcate method for the identification of differentially methylated DNA regions; Additional Tables listing the genes and number of CpG sites investigated for differential methylation between former PICU patients and healthy children, the differentially methylated regions in former PICU patients versus healthy children, the interaction of differential methylation in former PICU patients versus healthy children with sex and age at exposure, and the analyses of differential methylation between former PICU patients who received glucocorticoids during their stay in the PICU versus those who did not; and Additional Figure summarising a speculative interpretation of potential impact of abnormal DNA methylation within steroidogenic genes on corresponding gene expression. [file 13148_2023_1530_MOESM1_ESM.pdf]

Abnormal DNA methylation within genes of the steroidogenesis pathway two years after paediatric critical illness and association with stunted growth in height further in time

Ilse Vanhorebeek, MEng, PhD[1*], Grégoire Coppens, MD[1*], Fabian Güiza, MEng, PhD[1], Inge Derese BSc[1], Pieter J Wouters, MSc[1], Koen F. Joosten, MD, PhD[2], Sascha C. Verbruggen, MD, PhD[2], Greet Van den Berghe, MD, PhD[1]

* Contributed equally

[1] Clinical Division and Laboratory of Intensive Care Medicine, Department of Cellular and Molecular Medicine, KU Leuven, Leuven, Belgium; [2] Division of Paediatric ICU, Department of Neonatal and Paediatric ICU, Erasmus Medical Centre, Sophia Children's Hospital, Rotterdam, The Netherlands.

Corresponding author: Greet Van den Berghe, Clinical Division and Laboratory of Intensive Care Medicine, KU Leuven, Herestraat 49, B-3000 Leuven, Belgium. Phone: 32-16-34-40-21; Fax: 32-16-34-40-15; Email: greet.vandenberghe@med.kuleuven.be; ORCID: 0000-0002-5320-1362

ADDITIONAL FILE 1

Methods A1: Definition of "Syndrome"

Methods A2: Stepwise explanation of the DMRcate method for the identification of differentially methylated DNA regions

Table A1: Genes and number of CpG sites investigated for differential methylation between former PICU patients and healthy children

Table A2: CpG sites investigated for differential methylation between former PICU patients and healthy children

Table A3: Differentially methylated regions in former PICU patients versus healthy children

Table A4: Interaction of differential methylation in former PICU patients versus healthy children with sex

Table A5: Interaction of differential methylation in former PICU patients versus healthy children with age at exposure

Table A6: Differentially methylated CpG sites between former PICU patients who received glucocorticoids during their stay in the PICU versus those who did not

Figure A1: Speculative interpretation of potential impact of abnormal DNA methylation within steroidogenic genes on corresponding gene expression

Methods A1: Definition of “Syndrome”

A pre-randomisation syndrome or illness *a priori* defined as affecting or possibly affecting neurocognitive development, and which is subdivided in the following categories:

- Genetically confirmed syndrome or pathogenic chromosomal abnormality
- Clearly defined syndrome, association or malformation without (identified) genetic aberration
- Polymalformative syndrome of unknown aetiology
- Clear auditory or visual impairment without specified syndrome
- Congenital hypothyroidism due to thyroid agenesis
- Brain tumour or tumour with intracranial metastatic disease
- Paediatric psychiatric disorder (e.g. autism spectrum disorder, (treatment for) attention deficit hyperactivity disorder)
- Severe medical disorder, not primarily neurologic, but suspected to alter psychomotor and/or mental performance
- Severe neonatal problem (e.g. severe asphyxia)
- Severe craniocerebral trauma or near-drowning
- Severe infectious encephalitis or drug-induced encephalopathy
- Infectious meningitis, encephalitis or Guillain-Barré
- Resuscitation and/or need for extracorporeal membrane oxygenation prior to randomisation
- Severe convulsions or stroke prior to randomisation

Methods A2: Stepwise explanation of the DMRcate method for the identification of differentially methylated DNA regions

Due to the complexity of the 'dmrcate' method, we here illustrate this technique in a simplified stepwise manner, using an example. The data used in this theoretical example are for illustrative purposes only.

Step 1: Take the squared moderated t-statistic from the multivariable linear regression models that were used to identify the DMPs.

Step 2: Calculate the kernel estimate for every location in the DNA that contains a CpG site. A kernel estimate is the weighted sum of all the moderated t^2 statistics in a given chromosome. Weights are defined by the distance from the location of interest using gaussian smoothing. Weights decrease with increasing distance to the CpG site of interest.

As demonstrated in the figure below, the kernel estimate of CpG 6 can be computed as follows (<u>underlined</u>: the t-statistic, bold: the kernel weight based on the distance from the CpG 6):

Weighted sum: <u>4.55</u>***0.01** + <u>3.22</u>***0.05** + … + <u>2.13</u>***0.29** + <u>7.37</u>***1.00** + <u>6.12</u>***0.18** + … + <u>5.89</u>***0.08** = 10.56

Step 3: Calculate the p-value of every kernel estimate against a null hypothesis of no methylation difference. In the example below, statistically significant kernel estimates are denoted in green.

Step 4: Agglomerate statistically significant kernel estimates that are not further then 1000 BP (λ) apart. This stepwise explanation is a simplified version of the original method, for more information on how the weights of the kernel estimates are calculated or how the p-values are calculated, we refer to the work from Peter et al [A1].

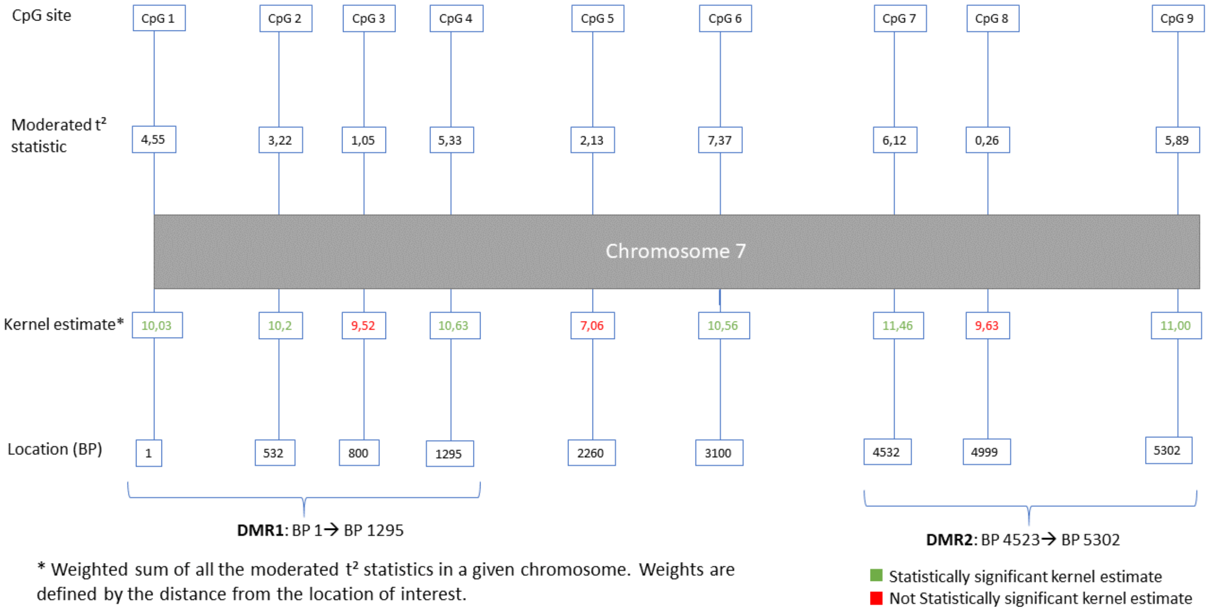
CpG site
CpG 1
CpG 2
CpG 3
CpG 4
CpG 5
CpG 6
CpG 7
CpG 8
CpG 9
Moderated t² statistic
4,55
3,22
1,05
5,33
2,13
7,37
6,12
0,26
5,89
Chromosome 7
Kernel estimate*
10,03
10,2
9,52
10,63
7,06
10,56
11,46
9,63
11,00
Location (BP)
1
532
800
1295
2260
3100
4532
4999
5302
DMR1: BP 1→ BP 1295
DMR2: BP 4523→ BP 5302
* Weighted sum of all the moderated t² statistics in a given chromosome. Weights are defined by the distance from the location of interest.
Statistically significant kernel estimate
Not Statistically significant kernel estimate

Table A1: Genes and number of CpG sites investigated for differential methylation between former PICU patients and healthy children

Gene name	Number of CpG sites	Function/Enzymatic activity
CYP11A1	37	Cholesterol → pregnenolone
FDX1	20	Involved in transport of electrons to CYP11A1
FDX2	19	Involved in transport of electrons to CYP11A1
FDXR	22	Involved in transport of electrons to CYP11A1
CYP17A1	10	Pregnenolone → 17OH-pregnenolone → dehydroepiandrosterone<br>Progesterone → 17OH-progesterone (→ androstenedione)
POR	69	Involved in transport of electrons to CYP17A1
CYB5A	28	Involved in transport of electrons to CYP17A1
CYB5B	27	Involved in transport of electrons to CYP17A1
HSD3B1/<br>HSD3B2	6/8	Pregnenolone → progesterone, 17OH-pregnenolone → 17OH-progesterone, dehydroepiandrosterone → androstenedione, androstenediol → testosterone
HSD17B1	15	Estrone → estradiol, inactivation of dihydrotestosterone<br>(dehydroepiandrosterone → androstenediol),<br>(androstenedione → testosterone)
HSD17B2	32	Androstenediol → dehydroepiandrosterone, testosterone → androstenedione, estradiol → estrone
HSD17B3	19	Androstenedione → testosterone
HSD17B6	10	Estradiol → estrone<br>(3alpha-adiol → dihydrotestosterone, androsterone → epiandrosterone)<br>(5alpha-androstan-3alpha,17beta-diol → androsterone)
HSD17B7	13	Estrone → estradiol, inactivation of dihydrotestosterone
HSD17B8	36	Estradiol → estrone<br>(testosterone → androstenedione, inactivation dihydrotestosterone)
HSD17B10	15	Estradiol → estrone, 3alpha-androstanediol → dihydrotestosterone
HSD17B11	18	Androstan-3-alpha,17-beta-diol (3-alpha-diol) →androsterone (in vitro)
HSD17B12	47	Estrone → estradiol
HSD17B14	16	Estradiol → estrone
CYP19A1	55	Androstenedione → estrone, testosterone → estradiol
CYP21A2	13	Progesterone → 11-deoxycorticosterone,<br>17OH-progesterone → 11-deoxycortisol
CYP11B1	13	11-deoxycorticosterone → corticosterone,<br>11-deoxycortisol → cortisol
CYP11B2	11	11-deoxycorticosterone → corticosterone → 18OH-corticosterone → aldosterone
STS	17	Steroid sulfatase
SULT1E1	5	Sulfotransferase, sulfates estrone
SULT2A1	10	Sulfotransferase, sulfates steroids
SULT2B1	36	Sulfotransferase, sulfates dehydroepiandrosterone

Enzyme reactions indicated between brackets refer to weaker reactions/less preferred substrates.

Table A2: CpG sites investigated for differential methylation between former PICU patients and healthy children

Gene	CpG	Chromosome	Position [a]	Gene section [b,c]	Log fold change [d]	P [e]	FANTOM4 Enhancer location [f]	FANTOM5 Enhancer location [g]	ENCODE Regulatory feature location [h]
CYP11A1	cg03449379	chr15	74630237	3'UTR	0.061 [-0.025 – 0.148]	0.39			15:74630188-74630301
	cg06457577	chr15	74631030	Body	-0.006 [-0.050 – 0.039]	0.94			
	cg10970701	chr15	74631708	Body	-0.015 [-0.054 – 0.025]	0.73			
	cg18630275	chr15	74632092	Body/NA	-0.006 [-0.046 – 0.034]	0.92			
	cg04971286	chr15	74636264	Body	-0.038 [-0.065 – -0.011]	0.092			
	cg21573592	chr15	74636274	Body	-0.031 [-0.084 – 0.022]	0.52			
	cg04874865	chr15	74636306	Body	-0.008 [-0.046 – 0.030]	0.86			
	cg12931909	chr15	74637261	Body	-0.026 [-0.055 – 0.003]	0.24			
	cg14349956	chr15	74637483	Body	-0.046 [-0.079 – -0.012]	0.095			
	cg15673413	chr15	74645437	5'UTR/Body	0.006 [-0.035 – 0.047]	0.92			
	cg03630056	chr15	74645873	5'UTR/Body	-0.052 [-0.097 – -0.008]	0.13			
	cg12387434	chr15	74652543	5'UTR/Body	0.026 [-0.023 – 0.076]	0.56			
	cg20491017	chr15	74656876	5'UTR/Body	0.012 [-0.038 – 0.062]	0.83			
	cg06837499	chr15	74656895	5'UTR/Body	0.010 [-0.025 – 0.046]	0.81			
	cg25788983	chr15	74657584	5'UTR/Body	0.099 [0.017 – 0.181]	0.12			
	cg05454635	chr15	74657911	5'UTR/Body	0.072 [-0.013 – 0.157]	0.28			
	cg09490603	chr15	74658166	5'UTR/1stExon/Body	0.010 [-0.045 – 0.065]	0.89			15:74658144-74658598
	cg02916102	chr15	74658244	5'UTR/1stExon/Body	0.012 [-0.018 – 0.041]	0.70			15:74658144-74658598
	cg16332610	chr15	74658547	5'UTR/1stExon/Body	-0.010 [-0.056 – 0.037]	0.87			15:74658144-74658598
	cg14855334	chr15	74658553	Promoter/Body	0.010 [-0.017 – 0.037]	0.73			15:74658144-74658598
	cg24578679	chr15	74658573	Promoter/Body	0.000 [-0.031 – 0.031]	0.99			15:74658144-74658598
	cg17794241	chr15	74658653	Promoter/Body	0.021 [-0.005 – 0.047]	0.31			
	cg06384523	chr15	74658693	Promoter/Body	-0.019 [-0.057 – 0.019]	0.60			
	cg00615746	chr15	74658981	Promoter/Body	-0.019 [-0.091 – 0.053]	0.82			
	cg07131601	chr15	74659404	Promoter/Body	-0.024 [-0.052 – 0.004]	0.27			
	cg23808031	chr15	74659841	Promoter/1stExon	0.195 [0.090 – 0.301]	0.016			
	cg06285340	chr15	74659901	Promoter/1stExon	0.062 [0.004 – 0.121]	0.17			

	cg08033551	chr15	74659966	Promoter/5'UTR/1stExon	0.127 [0.009 – 0.244]	0.17		
	cg23660703	chr15	74660059	5'UTR/1stExon	0.098 [-0.008 – 0.205]	0.23		
	cg18068537	chr15	74660088	Promoter	0.120 [0.027 – 0.213]	0.10		
	cg05939495	chr15	74660110	Promoter	0.112 [-0.003 – 0.228]	0.20		
	cg02137675	chr15	74660142	Promoter	0.095 [0.009 – 0.181]	0.16		
	cg17790333	chr15	74660265	Promoter	-0.007 [-0.046 – 0.032]	0.89		
	cg20330630	chr15	74660375	Promoter	-0.013 [-0.069 – 0.044]	0.85		
	cg15151764	chr15	74660563	Promoter	0.014 [-0.082 – 0.111]	0.92		
	cg05944462	chr15	74661351	Promoter	-0.030 [-0.075 – 0.015]	0.45		
	cg24849517	chr15	74661541	Promoter	-0.003 [-0.042 – 0.037]	0.97		
FDX1	cg10414747	chr11	110299159	Promoter	-0.016 [-0.099 – 0.068]	0.89		
	cg06674932	chr11	110299342	Promoter	-0.010 [-0.050 – 0.030]	0.83		
	cg00732589	chr11	110299881	Promoter	-0.069 [-0.130 – -0.007]	0.15		
	cg05741490	chr11	110300161	Promoter	-0.090 [-0.160 – -0.020]	0.10		
	cg26763524	chr11	110300429	Promoter	0.033 [-0.008 – 0.073]	0.30		
	cg08887425	chr11	110300464	Promoter	-0.004 [-0.044 – 0.036]	0.95		
	cg26818187	chr11	110300471	Promoter	0.025 [-0.014 – 0.065]	0.46		
	cg18719294	chr11	110300575	Promoter	0.006 [-0.026 – 0.038]	0.89		11:110300503-110301225
	cg09762563	chr11	110300584	Promoter	-0.005 [-0.065 – 0.055]	0.97		11:110300503-110301225
	cg13258606	chr11	110300632	5'UTR/1stExon	0.016 [-0.018 – 0.050]	0.63	high-CpG:109805799-109805924	11:110300503-110301225
	cg02525171	chr11	110300675	5'UTR/1stExon	0.028 [0.007 – 0.049]	0.10	high-CpG:109805799-109805924	11:110300503-110301225
	cg23587050	chr11	110301128	Body	0.001 [-0.027 – 0.028]	0.98		11:110300503-110301225
	cg05485370	chr11	110301333	Body	0.015 [-0.028 – 0.057]	0.75		
	cg20364660	chr11	110301606	Body	0.041 [0.008 – 0.074]	0.11		11:110301456-110302247
	cg26061355	chr11	110304169	Body	-0.028 [-0.067 – 0.010]	0.37		11:110303854-110306626
	cg08911148	chr11	110308998	Body	-0.023 [-0.081 – 0.035]	0.70		
	cg10527683	chr11	110317131	Body	-0.031 [-0.071 – 0.009]	0.32		
	cg24790674	chr11	110324793	Body	-0.018 [-0.051 – 0.015]	0.56		
	cg12584858	chr11	110326628	Body	0.169 [0.039 – 0.299]	0.10		
	cg02239377	chr11	110334097	3'UTR	0.009 [-0.026 – 0.044]	0.82		11:110333213-110334533

FDX2	cg03891588	chr19	10420902	3'UTR	-0.009 [-0.041 – 0.024]	0.82	
	cg12963297	chr19	10421057	3'UTR	0.014 [-0.019 – 0.047]	0.68	
	cg15639464	chr19	10421076	3'UTR	-0.019 [-0.053 – 0.015]	0.55	
	cg13507706	chr19	10421712	Body	-0.005 [-0.037 – 0.026]	0.90	
	cg14572892	chr19	10424068	Body	-0.029 [-0.065 – 0.006]	0.30	
	cg23247595	chr19	10426110	Body	-0.003 [-0.041 – 0.035]	0.97	19:10426078-10427272
	cg14151616	chr19	10426492	Body	0.018 [-0.005 – 0.042]	0.32	19:10426078-10427272
	cg02764611	chr19	10426516	Body	0.009 [-0.027 – 0.044]	0.83	19:10426078-10427272
	cg27627560	chr19	10426574	1stExon	0.000 [-0.037 – 0.037]	0.99	19:10426078-10427272
	cg05082703	chr19	10426711	Promoter	0.002 [-0.037 – 0.042]	0.97	19:10426078-10427272
	cg00791249	chr19	10426722	Promoter	0.000 [-0.040 – 0.040]	0.99	19:10426078-10427272
	cg16431088	chr19	10426777	Promoter	0.005 [-0.038 – 0.048]	0.94	19:10426078-10427272
	cg27101112	chr19	10426802	Promoter	0.042 [0.007 – 0.077]	0.12	19:10426078-10427272
	cg03670835	chr19	10426826	Promoter	-0.013 [-0.050 – 0.024]	0.74	19:10426078-10427272
	cg02456552	chr19	10426933	Promoter	0.007 [-0.025 – 0.04]	0.85	19:10426078-10427272
	cg27259408	chr19	10427154	Promoter	0.069 [0.021 – 0.117]	0.083	19:10426078-10427272
	cg04701317	chr19	10427639	Promoter	-0.016 [-0.045 – 0.013]	0.54	
	cg21716560	chr19	10428088	Promoter	0.035 [-0.004 – 0.073]	0.24	19:10428039-10428112
	cg27181254	chr19	10428163	Promoter	-0.015 [-0.049 – 0.019]	0.65	
FDXR	cg25268537	chr17	72860402	Body	-0.026 [-0.066 – 0.013]	0.43	17:72860219-72861513
	cg02461739	chr17	72860448	Body	-0.027 [-0.059 – 0.005]	0.29	17:72860219-72861513
	cg02065484	chr17	72864357	Body	-0.019 [-0.050 – 0.011]	0.47	
	cg14007128	chr17	72864722	5'UTR/1stExon/Body	-0.055 [-0.094 – -0.016]	0.092	
	cg25251853	chr17	72865942	Promoter/Body	-0.012 [-0.044 – 0.019]	0.71	
	cg27464296	chr17	72866030	Body	0.006 [-0.029 – 0.042]	0.89	

	cg11606825	chr17	72866125	Promoter/Body	-0.024 [-0.055 – 0.008]	0.35	
	cg01883267	chr17	72866175	Promoter/Body	-0.013 [-0.039 – 0.014]	0.63	
	cg00039279	chr17	72866822	Body	0.003 [-0.072 – 0.079]	0.98	
	cg24737900	chr17	72869117	5'UTR/1stExon	-0.017 [-0.044 – 0.011]	0.50	17:72868458-72870310
	cg11769049	chr17	72869140	5'UTR/1stExon	0.028 [-0.002 – 0.058]	0.22	17:72868458-72870310
	cg23273911	chr17	72869156	Promoter	0.017 [-0.013 – 0.048]	0.53	17:72868458-72870310
	cg16828937	chr17	72869164	Promoter	0.025 [-0.010 – 0.059]	0.38	17:72868458-72870310
	cg00904063	chr17	72869185	Promoter	0.017 [-0.012 – 0.047]	0.52	17:72868458-72870310
	cg11140440	chr17	72869219	Promoter	0.038 [0.002 – 0.074]	0.17	17:72868458-72870310
	cg08112448	chr17	72869226	Promoter	0.031 [-0.006 – 0.069]	0.29	17:72868458-72870310
	cg09841758	chr17	72869237	Promoter	0.025 [-0.016 – 0.066]	0.50	17:72868458-72870310
	cg07917836	chr17	72869660	Promoter	0.027 [-0.016 – 0.069]	0.47	17:72868458-72870310
	cg18061678	chr17	72869702	Promoter	-0.001 [-0.034 – 0.032]	0.98	17:72868458-72870310
	cg02501186	chr17	72869935	Promoter	0.025 [-0.003 – 0.053]	0.24	17:72868458-72870310
	cg16376748	chr17	72870102	Promoter	-0.027 [-0.062 – 0.007]	0.32	17:72868458-72870310
	cg25786980	chr17	72870188	Promoter	0.034 [-0.019 – 0.088]	0.47	17:72868458-72870310
CYP17A1	cg10756593	chr10	104591244	Body/NA	0.005 [-0.035 – 0.045]	0.94	
	cg27496231	chr10	104591314	Body	0.010 [-0.050 – 0.069]	0.91	
	cg24525156	chr10	104592367	Body	0.002 [-0.043 – 0.048]	0.97	
	cg17932736	chr10	104592419	Body/NA	-0.019 [-0.060 – 0.022]	0.64	
	cg07809032	chr10	104592955	Body/NA	-0.001 [-0.031 – 0.028]	0.97	
	cg09201719	chr10	104596890	1stExon	-0.011 [-0.045 – 0.024]	0.79	
	cg10127942	chr10	104597661	Promoter	0.019 [-0.022 – 0.059]	0.63	
	cg24934431	chr10	104597864	Promoter	-0.030 [-0.090 – 0.029]	0.59	
	cg15595910	chr10	104597896	Promoter	-0.017 [-0.040 – 0.006]	0.35	
	cg09729955	chr10	104598378	Promoter	-0.025 [-0.069 – 0.020]	0.55	

POR	cg14016166	chr7	75543425	Promoter	0.020 [-0.065 – 0.105]	0.84			7:75543417-75543644
	cg18773807	chr7	75543705	Promoter	0.002 [-0.072 – 0.075]	0.98			
	cg05435065	chr7	75544030	Promoter	0.027 [-0.014 – 0.068]	0.45			7:75543820-75545572
	cg12348198	chr7	75544203	Promoter	-0.016 [-0.053 – 0.022]	0.69			7:75543820-75545572
	cg05494152	chr7	75544339	Promoter	0.008 [-0.022 – 0.038]	0.82			7:75543820-75545572
	cg07961444	chr7	75544359	Promoter	0.014 [-0.009 – 0.037]	0.49			7:75543820-75545572
	cg18219712	chr7	75544485	5'UTR/1stExon	0.010 [-0.003 – 0.050]	0.83	high-CpG:75382375-75382571		7:75543820-75545572
	cg17488228	chr7	75544515	5'UTR	0.020 [-0.002 – 0.042]	0.23	high-CpG:75382375-75382571		7:75543820-75545572
	cg15699691	chr7	75545455	5'UTR	0.096 [0.014 – 0.177]	0.13			7:75543820-75545572
	cg04374289	chr7	75547905	5'UTR	-0.021 [-0.077 – 0.036]	0.73			
	cg17813164	chr7	75550364	5'UTR	-0.014 [-0.042 – 0.015]	0.62			
	cg10038278	chr7	75551428	5'UTR	-0.004 [-0.046 – 0.038]	0.95			
	cg12318049	chr7	75552312	5'UTR	0.208 [0.047 – 0.368]	0.10			
	cg11607004	chr7	75556324	5'UTR	0.173 [0.041 – 0.306]	0.10			
	cg08466464	chr7	75557956	5'UTR	0.018 [-0.055 – 0.091]	0.83			7:75557855-75558351
	cg01216063	chr7	75561005	5'UTR	-0.031 [-0.067 – 0.005]	0.28			
	cg11520834	chr7	75563746	5'UTR	-0.003 [-0.030 – 0.024]	0.94		chr7:75563701-75563701	
	cg15177211	chr7	75567469	5'UTR	-0.064 [-0.178 – 0.050]	0.53		chr7:75567331-75567331	
	cg02138834	chr7	75567608	5'UTR	-0.018 [-0.054 – 0.018]	0.59		chr7:75567331-75567331	
	cg05250352	chr7	75571631	5'UTR	0.030 [-0.052 – 0.111]	0.73			
	cg12278697	chr7	75572817	5'UTR	-0.022 [-0.066 – 0.022]	0.59			
	cg10902139	chr7	75573071	5'UTR	0.113 [-0.018 – 0.245]	0.27			
	cg01657188	chr7	75573154	5'UTR	-0.021 [-0.059 – 0.016]	0.53			
	cg10738873	chr7	75579540	5'UTR	-0.116 [-0.175 – -0.058]	0.0092			
	cg17115737	chr7	75580813	5'UTR	-0.099 [-0.159 – -0.039]	0.036			
	cg22898055	chr7	75581161	5'UTR	0.005 [-0.040 – 0.049]	0.95		chr7:75580822-75580822	

cg20720686	chr7	75582881	5'UTR	-0.140 [-0.252 – -0.029]	0.11		
cg22633023	chr7	75583185	5'UTR	-0.109 [-0.208 – -0.010]	0.16		
cg06556337	chr7	75583334	Body	-0.006 [-0.038 – 0.027]	0.90		
cg20748065	chr7	75583421	Body	-0.170 [-0.297 – -0.043]	0.10		
cg01676795	chr7	75586348	Body	0.108 [-0.005 – 0.221]	0.21		
cg01890085	chr7	75587983	Body	-0.019 [-0.067 – 0.030]	0.71		
cg15983105	chr7	75590671	Body	-0.007 [-0.045 – 0.031]	0.89		
cg16182457	chr7	75592134	Body	-0.002 [-0.055 – 0.051]	0.98		
cg27342333	chr7	75592418	Body	-0.062 [-0.121 – -0.003]	0.18		
cg18630265	chr7	75592838	Body	-0.058 [-0.111 – -0.005]	0.16		
cg14500655	chr7	75595148	Body	0.005 [-0.042 – 0.052]	0.95		
cg14204559	chr7	75596021	Body	-0.030 [-0.062 – 0.001]	0.20	chr7:75595933-75595933	
cg21840908	chr7	75596046	Body	0.033 [-0.012 – 0.079]	0.37	chr7:75595933-75595933	
cg18921306	chr7	75596055	Body	-0.033 [-0.102 – 0.037]	0.62	chr7:75595933-75595933	
cg22268049	chr7	75596207	Body	-0.003 [-0.049 – 0.044]	0.97	chr7:75595933-75595933	
cg26647549	chr7	75596992	Body	-0.076 [-0.162 – 0.011]	0.27		
cg12832057	chr7	75597068	Body	-0.026 [-0.089 – 0.037]	0.68		
cg03509253	chr7	75597100	Body	-0.058 [-0.111 – -0.005]	0.16		
cg26827455	chr7	75598687	Body	0.108 [0.008 – 0.207]	0.17	chr7:75598574-75598574	
cg27154660	chr7	75599137	Body	-0.022 [-0.053 – 0.009]	0.38		
cg07614473	chr7	75599701	Body	0.029 [-0.006 – 0.064]	0.29		
cg27372063	chr7	75601901	Body	-0.182 [-0.286 – -0.078]	0.026		7:75601477-75602026
cg16170009	chr7	75603430	Body	-0.044 [-0.085 – -0.004]	0.16		
cg21531916	chr7	75608553	Body	-0.019 [-0.068 – 0.029]	0.70		
cg02742533	chr7	75608829	Body	-0.016 [-0.053 – 0.022]	0.68		
cg18552347	chr7	75609532	Body	0.168 [0.003 – 0.333]	0.19		
cg14257191	chr7	75609719	Body	0.123 [-0.010 – 0.256]	0.23		
cg21748691	chr7	75609803	Body/NA	0.011 [-0.073 – 0.095]	0.93		
cg14695043	chr7	75610230	Body	-0.024 [-0.092 – 0.043]	0.74		
cg10038145	chr7	75610419	Body	-0.030 [-0.092 – 0.031]	0.60		
cg16766966	chr7	75610520	Body	0.031 [-0.037 – 0.099]	0.64		
cg21154876	chr7	75610913	Body	0.062 [-0.067 – 0.190]	0.62		
cg02727959	chr7	75611035	Body	0.023 [-0.104 – 0.151]	0.89		

	cg20220522	chr7	75611046	Body	0.057 [-0.046 – 0.159]	0.54		
	cg03135313	chr7	75612713	Body	-0.01 [-0.139 – 0.118]	0.97		
	cg13915028	chr7	75613107	Body	0.026 [-0.043 – 0.094]	0.72		
	cg05613015	chr7	75613149	Body	0.041 [-0.039 – 0.122]	0.58		
	cg05122910	chr7	75613194	Body	0.032 [-0.028 – 0.091]	0.56		
	cg05153729	chr7	75614406	Body	-0.026 [-0.070 – 0.018]	0.51		
	cg22567591	chr7	75614685	Body	-0.010 [-0.043 – 0.023]	0.79		
	cg18827685	chr7	75614882	Body/NA	0.029 [-0.017 – 0.075]	0.47		
	cg08262464	chr7	75615023	Body	-0.001 [-0.054 – 0.051]	0.98		
	cg16684958	chr7	75615977	3'UTR	-0.008 [-0.059 – 0.043]	0.92		
CYB5A	cg05403934	chr18	71923954	Body/3'UTR	0.004 [-0.042 – 0.050]	0.97		
	cg09181159	chr18	71928473	Body	-0.039 [-0.081 – 0.002]	0.22		
	cg22615078	chr18	71931546	Body	-0.025 [-0.056 – 0.006]	0.30		
	cg06486863	chr18	71932563	Body	0.016 [-0.017 – 0.048]	0.62		
	cg08273828	chr18	71933649	Body	-0.008 [-0.036 – 0.020]	0.81		
	cg18665422	chr18	71935066	Body	-0.007 [-0.042 – 0.029]	0.89		
	cg26834979	chr18	71935682	Body	-0.005 [-0.050 – 0.039]	0.94		
	cg22303600	chr18	71941181	Body	-0.009 [-0.045 – 0.027]	0.83		
	cg14574365	chr18	71948060	Body	0.006 [-0.036 – 0.048]	0.92		
	cg16924061	chr18	71955528	Body	-0.006 [-0.04 – 0.029]	0.92		
	cg13640874	chr18	71955599	Body	0.015 [-0.026 – 0.055]	0.74		
	cg21899520	chr18	71957896	Body	-0.036 [-0.119 – 0.047]	0.67		
	cg23685965	chr18	71958448	Body	0.092 [0.022 – 0.163]	0.10		18:71958299-71959985
	cg21951216	chr18	71958904	Body	0.037 [-0.003 – 0.077]	0.22		18:71958299-71959985
	cg10878998	chr18	71959092	1stExon	0.092 [0.018 – 0.166]	0.11		18:71958299-71959985
	cg06007607	chr18	71959170	5'UTR/1stExon	0.007 [-0.014 – 0.028]	0.78	high-CpG:70110120-70110214	18:71958299-71959985
	cg20256961	chr18	71959244	Promoter	0.012 [-0.009 – 0.033]	0.53		18:71958299-71959985
	cg23780514	chr18	71959249	Promoter	0.007 [-0.021 – 0.036]	0.83		18:71958299-71959985
	cg25481635	chr18	71959313	Promoter	0.024 [0.000 – 0.048]	0.20		18:71958299-71959985
	cg21413452	chr18	71959343	Promoter	-0.017 [-0.054 – 0.020]	0.64		18:71958299-71959985

	cg10882817	chr18	71959395	Promoter	-0.012 [-0.050 – 0.025]	0.77	18:71958299-71959985
	cg24304618	chr18	71959402	Promoter	0.015 [-0.011 – 0.040]	0.53	18:71958299-71959985
	cg07544653	chr18	71959407	Promoter	0.021 [0.000 – 0.042]	0.20	18:71958299-71959985
	cg27662877	chr18	71959595	Promoter	0.000 [-0.029 – 0.028]	0.98	18:71958299-71959985
	cg26549682	chr18	71959633	Promoter	0.021 [-0.015 – 0.057]	0.53	18:71958299-71959985
	cg22456192	chr18	71959684	Promoter	-0.015 [-0.050 – 0.019]	0.64	18:71958299-71959985
	cg05471090	chr18	71959795	Promoter	-0.034 [-0.077 – 0.009]	0.31	18:71958299-71959985
	cg18274065	chr18	71959833	Promoter	-0.133 [-0.196 – -0.071]	0.0067	18:71958299-71959985
CYB5B	cg07090074	chr16	69457218	Promoter	-0.050 [-0.110 – 0.009]	0.28	16:69457110-69457233
	cg11732247	chr16	69457334	Promoter	0.003 [-0.033 – 0.038]	0.97	
	cg02760167	chr16	69457337	Promoter	0.020 [-0.026 – 0.066]	0.65	
	cg14252136	chr16	69457645	Promoter	0.009 [-0.032 – 0.050]	0.87	
	cg07795548	chr16	69457652	Promoter	0.015 [-0.056 – 0.087]	0.86	
	cg07713807	chr16	69458177	Promoter	-0.008 [-0.041 – 0.025]	0.84	16:69458035-69458990
	cg03491503	chr16	69458309	Promoter	0.035 [-0.001 – 0.070]	0.20	16:69458035-69458990
	cg05439318	chr16	69458329	Promoter	-0.001 [-0.039 – 0.037]	0.98	16:69458035-69458990
	cg07707379	chr16	69458383	Promoter	-0.005 [-0.051 – 0.041]	0.94	16:69458035-69458990
	cg16659480	chr16	69458394	Promoter	-0.001 [-0.052 – 0.050]	0.98	16:69458035-69458990
	cg16376438	chr16	69458483	Promoter	0.008 [-0.024 – 0.040]	0.83	16:69458035-69458990
	cg08980645	chr16	69458511	5'UTR/1stExon	0.008 [-0.022 – 0.038]	0.82	16:69458035-69458990
	cg19135794	chr16	69458518	5'UTR/1stExon	0.021 [-0.009 – 0.051]	0.39	16:69458035-69458990

	cg00419333	chr16	69458555	5'UTR/1stExon	-0.002 [-0.034 – 0.030]	0.97		16:69458035-69458990
	cg22067472	chr16	69458746	1stExon	0.024 [-0.005 – 0.053]	0.30		16:69458035-69458990
	cg03912267	chr16	69458953	Body	0.004 [-0.031 – 0.040]	0.94		16:69458035-69458990
	cg02126497	chr16	69461571	Body	-0.037 [-0.074 – 0.000]	0.20		
	cg00450252	chr16	69463605	Body	0.004 [-0.034 – 0.043]	0.94		
	cg20270365	chr16	69470128	Body	0.006 [-0.031 – 0.044]	0.90		
	cg06667281	chr16	69472438	Body	-0.035 [-0.088 – 0.018]	0.45		
	cg24600366	chr16	69482837	Body	0.083 [-0.010 – 0.176]	0.25		
	cg15842160	chr16	69496391	Body	-0.001 [-0.028 – 0.026]	0.98		
	cg16410373	chr16	69496584	3'UTR	0.011 [-0.024 – 0.046]	0.78		
	cg23824987	chr16	69497556	3'UTR	-0.001 [-0.044 – 0.042]	0.98		
	cg24392473	chr16	69497645	3'UTR	-0.034 [-0.072 – 0.003]	0.24		
	cg09621896	chr16	69497698	3'UTR	-0.014 [-0.043 – 0.016]	0.63		
	cg04406620	chr16	69499075	3'UTR	-0.023 [-0.095 – 0.048]	0.77		
HSD3B1	cg16579646	chr1	120048352	Promoter	-0.013 [-0.048 – 0.021]	0.71		
	cg06409416	chr1	120048924	Promoter	-0.035 [-0.081 – 0.011]	0.34		
	cg19452916	chr1	120049076	Promoter	-0.024 [-0.056 – 0.008]	0.34		
	cg08611752	chr1	120049154	Promoter	-0.005 [-0.065 – 0.054]	0.97		
	cg16175792	chr1	120050104	Body	-0.012 [-0.050 – 0.026]	0.79		
	cg10675561	chr1	120052814	Body	0.005 [-0.069 – 0.080]	0.97	chr1:120052717-120052717	
HSD3B2	cg11843645	chr1	119956771	Promoter	-0.004 [-0.053 – 0.046]	0.97		
	cg04744660	chr1	119956846	Promoter	0.005 [-0.034 – 0.044]	0.94		
	cg27103975	chr1	119956916	Promoter	-0.068 [-0.137 – 0.001]	0.20		
	cg26051856	chr1	119957199	Promoter	-0.020 [-0.080 – 0.041]	0.78		
	cg20414217	chr1	119957380	Promoter	-0.004 [-0.054 – 0.046]	0.97		
	cg23476425	chr1	119957824	5'UTR/1stExon	-0.002 [-0.036 – 0.032]	0.97		
	cg04087608	chr1	119957969	5'UTR	-0.011 [-0.041 – 0.020]	0.74		
	cg09371351	chr1	119958041	5'UTR	-0.063 [-0.109 – -0.016]	0.099		
HSD17B1	cg06110326	chr17	40703212	Promoter	0.010 [-0.025 – 0.044]	0.81		
	cg21282998	chr17	40703429	Promoter	-0.007 [-0.033 – 0.019]	0.82		
	cg11510871	chr17	40703470	Promoter	-0.029 [-0.061 – 0.002]	0.22		
	cg16546489	chr17	40703702	Promoter	-0.010 [-0.045 – 0.024]	0.79		
	cg07553595	chr17	40703883	Promoter	-0.007 [-0.039 – 0.025]	0.85		
	cg26729026	chr17	40704463	5'UTR/1stExon	-0.021 [-0.049 – 0.006]	0.34		
	cg20404150	chr17	40705273	Body	0.019 [-0.045 – 0.083]	0.80		

	cg01612247	chr17	40705588	Body	0.211 [0.048 – 0.374]	0.10			17:40705450-40705778
	cg17035997	chr17	40705708	Body	0.199 [0.035 – 0.363]	0.12			17:40705450-40705778
	cg03086899	chr17	40706061	Body	0.028 [-0.011 – 0.067]	0.37			17:40705808-40707211
	cg15418287	chr17	40706385	Body	0.024 [-0.022 – 0.069]	0.57			17:40705808-40707211
	cg23622369	chr17	40706682	Body	0.032 [-0.038 – 0.102]	0.64	high-CpG:37960203-37960365		17:40705808-40707211
	cg02363277	chr17	40706718	Body	0.039 [0.015 – 0.062]	0.044	high-CpG:37960203-37960365		17:40705808-40707211
	cg23549869	chr17	40706776	Body	0.017 [-0.020 – 0.054]	0.63	high-CpG:37960203-37960365		17:40705808-40707211
	cg06848514	chr17	40707027	3'UTR	0.009 [-0.022 – 0.039]	0.82			17:40705808-40707211
HSD17B2	cg24628832	chr16	82067399	Promoter	-0.108 [-0.183 – -0.032]	0.086			
	cg09894383	chr16	82067445	Promoter	-0.080 [-0.126 – -0.035]	0.026			
	cg13875973	chr16	82068284	Promoter	-0.053 [-0.102 – -0.003]	0.17			
	cg22395539	chr16	82068567	Promoter	-0.109 [-0.207 – -0.011]	0.16			
	cg26479028	chr16	82068702	Promoter	-0.009 [-0.088 – 0.069]	0.94			
	cg11515282	chr16	82068797	Promoter	-0.100 [-0.148 – -0.052]	0.0067	low-CpG:80626229-80626503		
	cg20373326	chr16	82068803	Promoter	-0.135 [-0.190 – -0.080]	0.0009	low-CpG:80626229-80626503		
	cg19807685	chr16	82068980	5'UTR/1stExon	-0.124 [-0.182 – -0.065]	0.0067	low-CpG:80626229-80626503		
	cg26315602	chr16	82069046	1stExon	-0.073 [-0.113 – -0.034]	0.016			
	cg06555887	chr16	82069450	Body	-0.033 [-0.071 – 0.006]	0.28			16:82069418-82069501
	cg27395029	chr16	82069874	Body	-0.026 [-0.071 – 0.020]	0.53		chr16:82069809-82069809	
	cg07307142	chr16	82071433	Body	-0.093 [-0.154 – -0.031]	0.067		chr16:82071348-82071348	
	cg13994673	chr16	82078005	Body	-0.038 [-0.077 – 0.001]	0.20			
	cg08244303	chr16	82078460	Body	0.001 [-0.044 – 0.045]	0.98			
	cg06123435	chr16	82084892	Body	-0.014 [-0.046 – 0.018]	0.65			
	cg04867583	chr16	82089274	Body	-0.032 [-0.068 – 0.005]	0.27			
	cg15326304	chr16	82089946	Body	0.036 [-0.040 – 0.112]	0.63			

	cg25750099	chr16	82093373	Body	-0.027 [-0.058 – 0.004]	0.27	
	cg00365986	chr16	82096585	Body	-0.134 [-0.212 – -0.057]	0.026	
	cg05315365	chr16	82096597	Body	-0.170 [-0.259 – -0.082]	0.013	
	cg13740036	chr16	82096624	Body	-0.192 [-0.287 – -0.098]	0.0073	
	cg04654998	chr16	82097475	Body	-0.156 [-0.260 – -0.051]	0.067	
	cg16555157	chr16	82098262	Body	-0.010 [-0.050 – 0.030]	0.83	
	cg13535052	chr16	82100810	Body	-0.054 [-0.089 – -0.019]	0.061	
	cg10646020	chr16	82101686	Body	-0.012 [-0.054 – 0.029]	0.80	
	cg04561261	chr16	82104638	Body	0.043 [-0.067 – 0.153]	0.71	
	cg06704432	chr16	82115761	Body	0.007 [-0.050 – 0.065]	0.94	
	cg01224949	chr16	82117013	Body	-0.015 [-0.044 – 0.014]	0.57	
	cg01148073	chr16	82126267	Body	-0.010 [-0.054 – 0.034]	0.84	
	cg12514084	chr16	82126554	Body	-0.025 [-0.055 – 0.004]	0.28	
	cg09439754	chr16	82129088	Body	-0.046 [-0.105 – 0.013]	0.33	
	cg03416601	chr16	82132065	3'UTR	-0.013 [-0.045 – 0.019]	0.70	
HSD17B3	cg06800115	chr9	99001240	Body	-0.086 [-0.159 – -0.013]	0.13	
	cg11663699	chr9	99002226	Body	-0.044 [-0.090 – 0.002]	0.22	
	cg17076957	chr9	99006818	Body	0.007 [-0.042 – 0.055]	0.93	
	cg02377082	chr9	99007888	Body	0.003 [-0.043 – 0.049]	0.97	
	cg26687910	chr9	99008626	Body	-0.018 [-0.054 – 0.017]	0.59	
	cg13617035	chr9	99012757	Body	-0.034 [-0.066 – -0.002]	0.17	
	cg04736833	chr9	99013591	Body	-0.036 [-0.072 – -0.001]	0.19	
	cg16068729	chr9	99013717	Body/NA	0.010 [-0.026 – 0.046]	0.82	
	cg18014207	chr9	99015410	Body	-0.131 [-0.204 – -0.058]	0.025	
	cg03175967	chr9	99017184	Body	-0.024 [-0.058 – 0.010]	0.40	
	cg15368817	chr9	99018625	Body	0.006 [-0.082 – 0.095]	0.97	
	cg24030880	chr9	99019237	Body	0.066 [0.015 – 0.117]	0.10	
	cg05655575	chr9	99019743	Body	-0.040 [-0.081 – 0.000]	0.20	
	cg16811201	chr9	99035862	Body	-0.029 [-0.064 – 0.005]	0.28	
	cg14176638	chr9	99061672	Body	-0.045 [-0.081 – -0.010]	0.10	
	cg11554937	chr9	99064321	1stExon	-0.001 [-0.040 – 0.037]	0.98	
	cg14356530	chr9	99064450	Promoter	0.107 [0.024 – 0.190]	0.10	
	cg14633704	chr9	99065350	Promoter	-0.010 [-0.061 – 0.040]	0.87	
	cg11036615	chr9	99065757	Promoter	-0.003 [-0.048 – 0.042]	0.97	
HSD17B6	cg08932940	chr12	57155564	Promoter	-0.014 [-0.052 – 0.023]	0.71	
	cg24733036	chr12	57155643	Promoter	-0.011 [-0.049 – 0.027]	0.82	
	cg09997171	chr12	57155969	Promoter	-0.057 [-0.137 – 0.023]	0.38	
	cg21922731	chr12	57157130	5'UTR/1stExon	-0.044 [-0.069 – -0.018]	0.026	12:57156898-57157514

	cg26243277	chr12	57160673	5'UTR	-0.044 [-0.086 – -0.001]	0.19		
	cg04696393	chr12	57162488	5'UTR	-0.010 [-0.046 – 0.026]	0.82	chr12:57162340-57162340	
	cg13013807	chr12	57169617	Body	-0.001 [-0.075 – 0.074]	0.99		
	cg14312894	chr12	57171980	Body	0.008 [-0.020 – 0.036]	0.82		
	cg26348735	chr12	57178638	Body/NA	-0.002 [-0.033 – 0.028]	0.97		
	cg22064287	chr12	57181002	Body	0.059 [0.002 – 0.117]	0.18		
HSD11B7	cg07793952	chr1	162759343	Promoter	-0.030 [-0.054 – -0.007]	0.10		
	cg13377324	chr1	162759914	Promoter	-0.003 [-0.030 – 0.025]	0.95		
	cg04469686	chr1	162760199	Promoter	0.082 [-0.012 – 0.177]	0.27		1:162760114-162760473
	cg25373794	chr1	162760220	Promoter	0.110 [0.010 – 0.211]	0.16		1:162760114-162760473
	cg18107078	chr1	162760321	Promoter	0.099 [-0.009 – 0.207]	0.23		1:162760114-162760473
	cg07774025	chr1	162760659	Body	0.051 [-0.032 – 0.135]	0.50		1:162760546-162760692
	cg14779990	chr1	162763223	Body	-0.005 [-0.048 – 0.037]	0.94		
	ch.1.3188690F	chr1	162763344	Body	-0.005 [-0.036 – 0.025]	0.89		
	cg12985297	chr1	162766887	Body	-0.050 [-0.101 – 0.000]	0.20		
	cg23558885	chr1	162767159	Body	0.036 [-0.021 – 0.093]	0.47		
	cg21097091	chr1	162768715	Body	-0.003 [-0.030 – 0.023]	0.94		
	cg05349647	chr1	162770214	Body	0.016 [-0.031 – 0.063]	0.75		
	cg11164980	chr1	162782340	3'UTR	-0.008 [-0.040 – 0.024]	0.83		
HSD17B8	cg26750489	chr6	33171106	Promoter	-0.033 [-0.061 – -0.005]	0.13		
	cg10134509	chr6	33171319	Promoter	-0.018 [-0.057 – 0.021]	0.64		
	cg02362439	chr6	33171324	Promoter	-0.003 [-0.037 – 0.031]	0.97		
	cg25059165	chr6	33171467	Promoter	-0.012 [-0.039 – 0.016]	0.69		
	cg19388032	chr6	33171491	Promoter	-0.025 [-0.067 – 0.017]	0.50		
	cg15854333	chr6	33171765	Promoter	-0.022 [-0.055 – 0.011]	0.45		
	cg13747899	chr6	33171911	Promoter	-0.053 [-0.100 – -0.006]	0.15		
	cg16482654	chr6	33172200	Promoter	0.023 [-0.005 – 0.050]	0.30		6:33171945-33173651
	cg05507546	chr6	33172208	Promoter	0.029 [-0.045 – 0.103]	0.71		6:33171945-33173651
	cg15389490	chr6	33172333	Promoter	0.037 [0.009 – 0.065]	0.10		6:33171945-33173651
	cg24455405	chr6	33172353	Promoter	0.022 [-0.008 – 0.052]	0.35		6:33171945-33173651

cg22647810	chr6	33172371	Promoter	0.007 [-0.018 – 0.031]	0.82	6:33171945-33173651
cg09825713	chr6	33172388	Promoter	0.027 [-0.008 – 0.063]	0.33	6:33171945-33173651
cg12283120	chr6	33172398	Promoter	-0.016 [-0.057 – 0.024]	0.70	6:33171945-33173651
cg07433344	chr6	33172450	1stExon	0.007 [-0.021 – 0.036]	0.83	6:33171945-33173651
cg25141069	chr6	33172467	1stExon	0.026 [0.005 – 0.048]	0.12	6:33171945-33173651
cg03462975	chr6	33172517	Body	-0.030 [-0.063 – 0.003]	0.24	6:33171945-33173651
cg09483904	chr6	33172551	Body	0.012 [-0.017 – 0.041]	0.67	6:33171945-33173651
cg14460343	chr6	33172572	Body	0.019 [-0.046 – 0.085]	0.80	6:33171945-33173651
cg07817400	chr6	33172817	Body	-0.005 [-0.050 – 0.040]	0.94	6:33171945-33173651
cg00614834	chr6	33172869	Body	0.007 [-0.041 – 0.054]	0.92	6:33171945-33173651
cg09100940	chr6	33172902	Body	0.044 [0.002 – 0.086]	0.18	6:33171945-33173651
cg21555177	chr6	33173024	Body	0.062 [-0.011 – 0.135]	0.28	6:33171945-33173651
cg00225157	chr6	33173038	Body	0.087 [0.016 – 0.158]	0.12	6:33171945-33173651
cg01502872	chr6	33173119	Body	0.085 [0.015 – 0.156]	0.12	6:33171945-33173651
cg02802514	chr6	33173134	Body	0.079 [-0.003 – 0.161]	0.20	6:33171945-33173651
cg02647520	chr6	33173218	Body	0.000 [-0.044 – 0.044]	0.99	6:33171945-33173651
cg00155619	chr6	33173278	Body	0.120 [0.017 – 0.222]	0.13	6:33171945-33173651
cg11335171	chr6	33173333	Body	0.160 [0.040 – 0.279]	0.10	6:33171945-33173651
cg27018424	chr6	33173425	Body	0.150 [0.008 – 0.293]	0.18	6:33171945-33173651

	cg12699756	chr6	33173482	Body	0.154 [0.030 – 0.278]	0.11	6:33171945-33173651
	cg01970322	chr6	33173489	Body	0.145 [0.023 – 0.267]	0.13	6:33171945-33173651
	cg17066452	chr6	33173501	Body	0.106 [-0.004 – 0.217]	0.21	6:33171945-33173651
	cg04262934	chr6	33173581	Body	0.057 [-0.022 – 0.136]	0.37	6:33171945-33173651
	cg00640376	chr6	33173927	Body	-0.011 [-0.053 – 0.032]	0.83	
	cg25511278	chr6	33174168	Body	-0.041 [-0.099 – 0.018]	0.40	
HSD17B10	cg14253153	chrX	53458243	3'UTR	-0.009 [-0.068 – 0.050]	0.92	
	cg02883100	chrX	53460671	Body	0.097 [0.014 – 0.180]	0.13	X:53460499-53460929
	cg08255147	chrX	53460775	Body	0.100 [-0.007 – 0.208]	0.22	X:53460499-53460929
	cg04241572	chrX	53461213	Body	0.077 [0.018 – 0.135]	0.10	X:53461138-53461763
	cg21156383	chrX	53461271	1stExon	0.087 [0.003 – 0.172]	0.18	X:53461138-53461763
	cg26323797	chrX	53461386	Promoter	0.146 [0.062 – 0.231]	0.026	X:53461138-53461763
	cg01477427	chrX	53461421	Promoter	0.108 [0.038 – 0.178]	0.058	X:53461138-53461763
	cg02116333	chrX	53461433	Promoter	0.075 [0.001 – 0.149]	0.20	X:53461138-53461763
	cg00128197	chrX	53461455	Promoter	0.068 [0.008 – 0.129]	0.15	X:53461138-53461763
	cg20010130	chrX	53461462	Promoter	0.067 [0.006 – 0.129]	0.16	X:53461138-53461763
	cg24552529	chrX	53461540	Promoter	0.070 [-0.003 – 0.142]	0.21	X:53461138-53461763
	cg10018458	chrX	53461681	Promoter	0.033 [-0.037 – 0.103]	0.63	X:53461138-53461763
	cg14681882	chrX	53461688	Promoter	0.006 [-0.071 – 0.082]	0.97	X:53461138-53461763
	cg01349336	chrX	53461709	Promoter	0.014 [-0.057 – 0.084]	0.88	X:53461138-53461763
	cg01720332	chrX	53462721	Promoter	-0.007 [-0.041 – 0.027]	0.88	
HSD17B11	cg22608848	chr4	88258112	3'UTR	0.002 [-0.034 – 0.038]	0.97	

	cg02518222	chr4	88274874	Body	-0.049 [-0.157 – 0.058]	0.63	4:88274727-88275218
	cg12132765	chr4	88277464	Body	0.001 [-0.036 – 0.037]	0.98	
	cg03221563	chr4	88278465	Body	0.005 [-0.064 – 0.073]	0.97	
	cg23956017	chr4	88295085	Body	-0.009 [-0.044 – 0.026]	0.82	
	cg24918767	chr4	88297189	Body	0.006 [-0.056 – 0.068]	0.96	4:88296901-88297255
	cg15641998	chr4	88312323	5'UTR/1stExon	0.028 [-0.006 – 0.062]	0.30	4:88312045-88313002
	cg07021777	chr4	88312416	5'UTR/1stExon	0.032 [-0.009 – 0.073]	0.33	4:88312045-88313002
	cg26923905	chr4	88312455	Promoter	0.045 [0.014 – 0.076]	0.071	4:88312045-88313002
	cg23841344	chr4	88312519	Promoter	0.021 [-0.008 – 0.050]	0.37	4:88312045-88313002
	cg12370284	chr4	88312521	Promoter	0.014 [-0.011 – 0.039]	0.53	4:88312045-88313002
	cg00309650	chr4	88312547	Promoter	-0.015 [-0.057 – 0.028]	0.75	4:88312045-88313002
	cg22175561	chr4	88312553	Promoter	0.016 [-0.015 – 0.048]	0.58	4:88312045-88313002
	cg19795082	chr4	88312560	Promoter	0.018 [-0.014 – 0.049]	0.53	4:88312045-88313002
	cg22831514	chr4	88312706	Promoter	0.028 [0.000 – 0.057]	0.20	4:88312045-88313002
	cg01382180	chr4	88313064	Promoter	-0.002 [-0.037 – 0.033]	0.97	
	cg01626496	chr4	88313095	Promoter	-0.052 [-0.113 – 0.009]	0.28	
	cg12209505	chr4	88313666	Promoter	-0.019 [-0.053 – 0.014]	0.53	
HSD17B12	cg23575732	chr11	43701083	Promoter	-0.045 [-0.087 – -0.002]	0.18	
	cg07940794	chr11	43701299	Promoter	0.023 [-0.024 – 0.071]	0.61	
	cg03606954	chr11	43701349	Promoter	0.028 [-0.021 – 0.077]	0.53	
	cg06112137	chr11	43701433	Promoter	-0.010 [-0.045 – 0.025]	0.81	
	cg17725614	chr11	43701446	Promoter	-0.003 [-0.039 – 0.033]	0.97	
	cg11422045	chr11	43701985	Promoter	-0.004 [-0.043 – 0.034]	0.94	11:43701744-43703161
	cg11980147	chr11	43702080	Promoter	0.009 [-0.019 – 0.037]	0.78	11:43701744-43703161
	cg17585343	chr11	43702103	Promoter	0.005 [-0.016 – 0.026]	0.84	11:43701744-43703161

cg21212277	chr11	43702113	Promoter	-0.016 [-0.075 – 0.042]	0.82			11:43701744-43703161
cg10157203	chr11	43702128	Promoter	0.007 [-0.014 – 0.028]	0.75			11:43701744-43703161
cg04406808	chr11	43702245	5'UTR/1stExon	0.013 [-0.031 – 0.056]	0.81	high-CpG:43658813-43658932		11:43701744-43703161
cg26478074	chr11	43702299	5'UTR/1stExon	-0.001 [-0.043 – 0.041]	0.98	high-CpG:43658813-43658932		11:43701744-43703161
cg08999477	chr11	43702335	5'UTR/1stExon	0.025 [-0.006 – 0.056]	0.30	high-CpG:43658813-43658932		11:43701744-43703161
cg11948328	chr11	43702395	1stExon	0.028 [-0.007 – 0.062]	0.31			11:43701744-43703161
cg14244963	chr11	43702422	1stExon	0.025 [0.004 – 0.047]	0.13			11:43701744-43703161
cg27068170	chr11	43702737	Body	-0.002 [-0.039 – 0.034]	0.97			11:43701744-43703161
cg26786893	chr11	43705792	Body	-0.065 [-0.130 – 0.001]	0.20			
cg02262727	chr11	43707755	Body	0.003 [-0.023 – 0.029]	0.94			
cg10784983	chr11	43709197	Body	0.029 [-0.027 – 0.085]	0.58			
cg08292164	chr11	43711049	Body	-0.035 [-0.065 – -0.005]	0.14			
cg01364865	chr11	43715469	Body	-0.108 [-0.188 – -0.029]	0.095			
cg12297975	chr11	43719186	Body	0.013 [-0.027 – 0.052]	0.78			
cg21434342	chr11	43730450	Body	-0.046 [-0.085 – -0.008]	0.12			
cg09396832	chr11	43734267	Body	-0.032 [-0.125 – 0.060]	0.75			
cg02468154	chr11	43755634	Body	-0.034 [-0.060 – -0.009]	0.099			
cg17440014	chr11	43755653	Body	-0.008 [-0.038 – 0.021]	0.81			
cg14262884	chr11	43757533	Body	-0.081 [-0.131 – -0.030]	0.049			
cg14489013	chr11	43758349	Body	-0.063 [-0.127 – 0.000]	0.20			
cg12467123	chr11	43759361	Body	-0.023 [-0.043 – -0.004]	0.12			
cg02233915	chr11	43760352	Body	0.147 [0.048 – 0.247]	0.067			
cg12261604	chr11	43768895	Body	-0.113 [-0.225 – -0.001]	0.20			
cg06656616	chr11	43778387	Body	0.116 [0.016 – 0.216]	0.13			
cg21259720	chr11	43779382	Body	-0.002 [-0.032 – 0.028]	0.97		chr11:43779324-43779324	
cg21077321	chr11	43780652	Body	-0.133 [-0.212 – -0.055]	0.031			
cg27526577	chr11	43812279	Body	-0.046 [-0.113 – 0.021]	0.42			
cg16591985	chr11	43832714	Body	-0.020 [-0.057 – 0.017]	0.56			
cg23672769	chr11	43832733	Body	-0.011 [-0.054 – 0.031]	0.82			
cg03042467	chr11	43841652	Body	0.006 [-0.065 – 0.078]	0.97			

	cg14757747	chr11	43846857	Body	0.076 [0.019 – 0.132]	0.10		
	cg14753094	chr11	43852883	Body	0.194 [0.066 – 0.321]	0.065		
	cg26734908	chr11	43856336	Body	0.000 [-0.030 – 0.029]	0.99		
	cg08271705	chr11	43856828	Body	0.204 [0.038 – 0.370]	0.12		
	cg16540215	chr11	43856925	Body	0.165 [0.035 – 0.295]	0.11		
	cg12933455	chr11	43859495	Body	-0.016 [-0.042 – 0.010]	0.50		
	cg04936289	chr11	43873567	Body	-0.024 [-0.054 – 0.006]	0.30		
	cg23470083	chr11	43873618	Body	0.023 [-0.008 – 0.053]	0.35		
	cg24324572	chr11	43876846	3'UTR	-0.013 [-0.049 – 0.023]	0.74		
HSD17B14	cg19046189	chr19	49316732	Body	-0.045 [-0.082 – -0.008]	0.12		19:49316691-49317073
	cg08054901	chr19	49318444	Body	0.012 [-0.026 – 0.050]	0.79		
	cg21745165	chr19	49319447	Body	-0.042 [-0.115 – 0.032]	0.53		
	cg17070116	chr19	49322937	Body	0.087 [0.009 – 0.164]	0.15		
	cg18753364	chr19	49326203	Body	-0.019 [-0.051 – 0.013]	0.50		
	cg04703666	chr19	49330003	Body	-0.075 [-0.155 – 0.004]	0.21		
	cg12181417	chr19	49337499	Body	-0.043 [-0.126 – 0.040]	0.58		
	cg07996880	chr19	49339085	Body	-0.015 [-0.065 – 0.035]	0.80	low-CpG:54030890-54030929	19:49338842-49339784
	cg24725522	chr19	49339397	Body	-0.089 [-0.196 – 0.019]	0.30		19:49338842-49339784
	cg16530498	chr19	49339776	5'UTR/1stExon	-0.033 [-0.086 – 0.020]	0.49		19:49338842-49339784
	cg15974053	chr19	49339789	5'UTR/1stExon	-0.040 [-0.104 – 0.024]	0.48		
	cg12326725	chr19	49340456	Promoter	-0.088 [-0.178 – 0.001]	0.20		19:49340102-49340931
	cg04777726	chr19	49340489	Promoter	-0.030 [-0.181 – 0.122]	0.88		19:49340102-49340931
	cg16594139	chr19	49340574	Promoter	-0.060 [-0.142 – 0.021]	0.36		19:49340102-49340931
	cg26267310	chr19	49340593	Promoter	-0.029 [-0.089 – 0.031]	0.61		19:49340102-49340931
	cg19024700	chr19	49340765	Promoter	-0.039 [-0.103 – 0.025]	0.50		19:49340102-49340931
CYP19A1	cg16122750	chr15	51501301	3'UTR	-0.022 [-0.069 – 0.024]	0.62		
	cg02325664	chr15	51507425	Body/NA	-0.036 [-0.093 – 0.020]	0.46		
	cg23719916	chr15	51508242	Body	-0.003 [-0.053 – 0.048]	0.97		
	cg15890210	chr15	51510841	Body/NA	-0.002 [-0.069 – 0.065]	0.98		
	cg07043857	chr15	51514698	Body	-0.016 [-0.043 – 0.011]	0.50		

cg03815549	chr15	51517028	Body	-0.001 [-0.032 – 0.030]	0.98			
cg12009872	chr15	51520739	Body	-0.057 [-0.092 – -0.021]	0.046	low-CpG:49307849-49308066		15:51520482-51520925
cg24203279	chr15	51522584	Body	0.017 [-0.033 – 0.067]	0.76			15:51522203-51522752
cg14424631	chr15	51526420	Body	-0.093 [-0.138 – -0.048]	0.0072			
cg03334874	chr15	51527087	Body	-0.009 [-0.045 – 0.027]	0.83			
cg00004322	chr15	51530189	Body	0.126 [-0.006 – 0.259]	0.21			
cg21478137	chr15	51532386	Body	-0.084 [-0.139 – -0.030]	0.058			
cg11285703	chr15	51534725	Body	0.000 [-0.051 – 0.051]	0.99			
cg01879211	chr15	51535382	5'UTR	0.011 [-0.035 – 0.057]	0.84			
cg08315265	chr15	51535432	5'UTR	0.078 [0.025 – 0.131]	0.067			
cg09577907	chr15	51535668	5'UTR	0.005 [-0.024 – 0.034]	0.90			
cg04083508	chr15	51535805	5'UTR	-0.005 [-0.039 – 0.030]	0.93			
cg08365349	chr15	51536845	5'UTR	-0.044 [-0.094 – 0.005]	0.25			
cg14156797	chr15	51537082	5'UTR	-0.049 [-0.116 – 0.018]	0.37			
cg19946085	chr15	51559439	5'UTR	0.021 [-0.014 – 0.056]	0.51			
cg06996165	chr15	51559577	5'UTR	0.024 [-0.020 – 0.067]	0.55			
cg12932492	chr15	51559826	5'UTR	0.021 [-0.016 – 0.057]	0.53			
cg09819222	chr15	51559977	5'UTR	0.021 [-0.021 – 0.063]	0.60			
cg09725032	chr15	51570474	5'UTR	-0.008 [-0.051 – 0.035]	0.88			
cg23397427	chr15	51571820	5'UTR	-0.032 [-0.072 – 0.008]	0.30			
cg17848615	chr15	51572136	5'UTR	-0.002 [-0.030 – 0.025]	0.97			
cg25814224	chr15	51572976	5'UTR	0.008 [-0.035 – 0.051]	0.89			15:51572693-51574342
cg24758719	chr15	51573197	5'UTR	-0.031 [-0.059 – -0.002]	0.17			15:51572693-51574342
cg13845570	chr15	51573586	5'UTR	-0.077 [-0.132 – -0.022]	0.092			15:51572693-51574342
cg23699588	chr15	51579699	5'UTR	-0.060 [-0.103 – -0.017]	0.092			
cg22267183	chr15	51581150	5'UTR	-0.017 [-0.058 – 0.024]	0.68			
cg14465057	chr15	51581239	5'UTR	0.000 [-0.028 – 0.027]	0.99			
cg14313646	chr15	51589654	5'UTR	-0.006 [-0.035 – 0.023]	0.87			
cg01916429	chr15	51590586	5'UTR	-0.019 [-0.073 – 0.034]	0.73		chr15:51590283-51590283	
cg15286970	chr15	51591447	5'UTR	-0.027 [-0.055 – 0.001]	0.20			
cg12330748	chr15	51596021	5'UTR	-0.053 [-0.088 – -0.019]	0.058			
cg19359072	chr15	51598433	5'UTR	-0.048 [-0.087 – -0.010]	0.11		chr15:51598187-51598187	

	cg12641629	chr15	51599332	5'UTR	0.076 [0.007 – 0.146]	0.16	
	cg04348026	chr15	51599844	5'UTR	0.187 [0.058 – 0.315]	0.073	
	cg24094625	chr15	51600409	5'UTR	-0.053 [-0.092 – -0.015]	0.095	
	cg19111801	chr15	51602474	5'UTR	0.001 [-0.056 – 0.059]	0.98	
	cg05492904	chr15	51604503	5'UTR	-0.166 [-0.296 – -0.037]	0.10	
	cg03144814	chr15	51607489	5'UTR	0.013 [-0.019 – 0.045]	0.70	
	cg12118187	chr15	51610734	5'UTR	0.014 [-0.022 – 0.049]	0.71	
	cg05852309	chr15	51611238	5'UTR	-0.002 [-0.047 – 0.043]	0.97	
	cg23028286	chr15	51614521	5'UTR	-0.048 [-0.094 – -0.002]	0.18	
	cg01666163	chr15	51616154	5'UTR	-0.003 [-0.031 – 0.025]	0.95	
	cg21792583	chr15	51616613	5'UTR	-0.015 [-0.051 – 0.022]	0.70	
	cg27467060	chr15	51619477	5'UTR	-0.006 [-0.037 – 0.024]	0.87	chr15:51619340-51619340
	cg20745775	chr15	51628372	5'UTR	-0.039 [-0.072 – -0.005]	0.14	
	cg15329467	chr15	51630696	5'UTR/1stExon	-0.016 [-0.046 – 0.014]	0.56	
	cg14694011	chr15	51631024	Promoter	0.023 [-0.02 – 0.066]	0.56	
	cg09205611	chr15	51631256	Promoter	-0.021 [-0.048 – 0.006]	0.34	
	cg20266498	chr15	51631495	Promoter	-0.028 [-0.055 – -0.001]	0.19	
	cg13216022	chr15	51631687	Promoter	-0.093 [-0.156 – -0.030]	0.069	
CYP21A2	cg03239580	chr6	31973051	Promoter	0.052 [-0.033 – 0.137]	0.50	
	cg11675917	chr6	31973162	Promoter	-0.129 [-0.199 – -0.060]	0.016	
	cg04771084	chr6	31973255	Promoter	0.005 [-0.072 – 0.082]	0.97	
	cg17404532	chr6	31973345	Promoter	-0.034 [-0.094 – 0.026]	0.53	
	cg14261401	chr6	31974906	Body	-0.021 [-0.063 – 0.022]	0.61	
	cg14616667	chr6	32005897	Promoter	-0.101 [-0.174 – -0.027]	0.095	
	cg27449793	chr6	32006074	Promoter	-0.028 [-0.071 – 0.015]	0.45	
	cg03157588	chr6	32006079	Promoter	-0.049 [-0.099 – 0.000]	0.20	
	cg18790646	chr6	32006196	5'UTR/1stExon	0.003 [-0.045 – 0.051]	0.97	
	cg08557799	chr6	32006951	Body	-0.009 [-0.045 – 0.026]	0.82	
	cg24487856	chr6	32007642	Body	0.003 [-0.041 – 0.047]	0.97	
	cg02953039	chr6	32007916	Body	-0.029 [-0.079 – 0.021]	0.53	
	cg12274082	chr6	32007986	Body	-0.041 [-0.087 – 0.005]	0.25	
CYP11B1	cg08389266	chr8	143955181	3'UTR	0.040 [-0.013 – 0.093]	0.35	
	cg14149685	chr8	143956517	Body	0.018 [-0.019 – 0.056]	0.61	
	cg26785106	chr8	143957347	Body	-0.033 [-0.067 – 0.000]	0.20	
	cg25376393	chr8	143957809	Body	-0.021 [-0.043 – 0.001]	0.20	
	cg20073007	chr8	143958385	Body	-0.007 [-0.031 – 0.018]	0.82	
	cg08230698	chr8	143958577	Body	0.010 [-0.053 – 0.072]	0.92	
	cg05416055	chr8	143960794	Body	-0.009 [-0.044 – 0.026]	0.83	

	cg02318454	chr8	143961141	1stExon	0.005 [-0.030 – 0.040]	0.92
	cg09120035	chr8	143961145	1stExon	-0.001 [-0.040 – 0.038]	0.98
	cg21901156	chr8	143961291	Promoter	0.031 [-0.011 – 0.072]	0.36
	cg03330425	chr8	143961303	Promoter	0.045 [-0.001 – 0.092]	0.20
	cg17015994	chr8	143961341	Promoter	0.009 [-0.028 – 0.047]	0.83
	cg01942530	chr8	143961514	Promoter	-0.014 [-0.058 – 0.029]	0.77
CYP11B2	cg15847600	chr8	143995000	Body	-0.046 [-0.082 – -0.009]	0.11
	cg02156704	chr8	143995688	Body/NA	0.056 [0.015 – 0.097]	0.095
	cg05210898	chr8	143997369	Body	0.026 [-0.027 – 0.079]	0.61
	cg02226239	chr8	143999155	1stExon	-0.030 [-0.060 – 0.001]	0.20
	cg07954324	chr8	143999330	Promoter	-0.001 [-0.032 – 0.030]	0.98
	cg11324259	chr8	143999374	Promoter	0.006 [-0.031 – 0.043]	0.92
	cg02467735	chr8	143999729	Promoter	-0.011 [-0.041 – 0.019]	0.72
	cg10500909	chr8	144000015	Promoter	-0.022 [-0.057 – 0.014]	0.50
	cg24155084	chr8	144000059	Promoter	-0.054 [-0.107 – -0.002]	0.18
	cg14389499	chr8	144000084	Promoter	-0.100 [-0.159 – -0.041]	0.031
	cg00136400	chr8	144000430	Promoter	0.003 [-0.048 – 0.054]	0.97
STS	cg09303788	chrX	7136404	Promoter	0.036 [-0.033 – 0.105]	0.58
	cg17460223	chrX	7136490	Promoter	0.179 [0.050 – 0.309]	0.092
	cg16232809	chrX	7136807	Promoter	-0.003 [-0.057 – 0.051]	0.97
	cg10985228	chrX	7136976	Promoter	0.109 [0.020 – 0.197]	0.12
	cg00596686	chrX	7137332	Promoter	-0.043 [-0.095 – 0.009]	0.30
	cg15027721	chrX	7137413	Promoter	0.069 [-0.012 – 0.150]	0.28
	cg01887803	chrX	7137474	5'UTR/1stExon	-0.022 [-0.076 – 0.032]	0.69
	cg19058005	chrX	7137529	5'UTR/1stExon	-0.002 [-0.047 – 0.044]	0.98
	cg11640565	chrX	7137913	Body	-0.047 [-0.098 – 0.003]	0.22
	cg10161978	chrX	7161004	Body	-0.027 [-0.063 – 0.008]	0.33
	cg14770642	chrX	7162390	Body	-0.048 [-0.106 – 0.010]	0.29
	cg25700851	chrX	7164772	Body	0.016 [-0.027 – 0.060]	0.72
	cg01468919	chrX	7165531	Body	-0.070 [-0.134 – -0.006]	0.16
	cg01129379	chrX	7180372	Body	-0.019 [-0.070 – 0.032]	0.73
	cg07478021	chrX	7182548	Body	0.028 [-0.021 – 0.077]	0.53
	cg04324977	chrX	7236373	Body	-0.024 [-0.064 – 0.016]	0.50
	cg10073470	chrX	7270088	3'UTR	0.080 [0.005 – 0.155]	0.17
SULT1E1	cg18336878	chr4	70709536	Body	0.008 [-0.048 – 0.065]	0.92
	cg02711994	chr4	70716021	Body	-0.024 [-0.068 – 0.020]	0.55
	cg14594278	chr4	70719125	Body	0.004 [-0.030 – 0.039]	0.94
	cg04895971	chr4	70724539	5'UTR	0.001 [-0.076 – 0.077]	0.99
	cg24746726	chr4	70726093	Promoter	-0.014 [-0.061 – 0.032]	0.79

SULT2A1	cg27488741	chr19	48374536	3'UTR	-0.022 [-0.123 – 0.079]	0.86		
	cg19774288	chr19	48385028	Body	-0.007 [-0.040 – 0.026]	0.85		
	cg03436935	chr19	48389435	1stExon	0.006 [-0.036 – 0.049]	0.92		
	cg19139729	chr19	48389562	5'UTR/1stExon	0.028 [-0.007 – 0.064]	0.31	low-CpG:53081365-53081408	
	cg22472023	chr19	48389615	5'UTR/1stExon	0.095 [0.003 – 0.187]	0.18		
	cg01554339	chr19	48389767	Promoter	0.017 [-0.032 – 0.065]	0.75		
	cg06181184	chr19	48389792	Promoter	0.001 [-0.030 – 0.033]	0.97		
	cg17013696	chr19	48389805	Promoter	0.033 [-0.013 – 0.079]	0.37		
	cg23835821	chr19	48389928	Promoter	-0.002 [-0.041 – 0.038]	0.98		
	cg26574395	chr19	48390114	Promoter	0.001 [-0.040 – 0.041]	0.98		
SULT2B1	cg18596640	chr19	49054612	Promoter	0.033 [-0.007 – 0.073]	0.29		
	cg18549547	chr19	49054908	Promoter	0.012 [-0.014 – 0.037]	0.64		
	cg12250421	chr19	49054962	Promoter	0.013 [-0.018 – 0.045]	0.68		
	cg15149098	chr19	49055027	Promoter	0.061 [-0.014 – 0.136]	0.30		
	cg10004595	chr19	49055211	Promoter	0.136 [0.017 – 0.254]	0.14		
	cg07108218	chr19	49055362	Promoter	0.182 [0.026 – 0.337]	0.13		
	cg03039843	chr19	49055390	Promoter	0.168 [0.028 – 0.307]	0.12		
	cg23097961	chr19	49055412	Promoter	0.129 [-0.011 – 0.268]	0.23		
	cg00698688	chr19	49055432	5'UTR/1stExon	0.175 [0.012 – 0.338]	0.17		
	cg08151612	chr19	49055438	5'UTR/1stExon	0.113 [-0.008 – 0.234]	0.22		
	cg07543967	chr19	49055443	5'UTR/1stExon	0.123 [0.001 – 0.244]	0.20		
	cg22967180	chr19	49057271	Body	0.149 [0.034 – 0.265]	0.10		
	cg04714466	chr19	49059513	Body	0.106 [0.004 – 0.209]	0.18		
	cg02314896	chr19	49059802	Body	0.012 [-0.037 – 0.061]	0.83		
	cg20117369	chr19	49061546	Body	0.100 [0.012 – 0.189]	0.15		
	cg05018821	chr19	49061676	Body	0.061 [0.005 – 0.117]	0.17		
	cg07869434	chr19	49061702	Body	0.096 [0.032 – 0.161]	0.067		
	cg27586378	chr19	49063066	Body	-0.013 [-0.041 – 0.014]	0.61		
	cg22629554	chr19	49063736	Body	0.002 [-0.031 – 0.035]	0.97		
	cg08363693	chr19	49063796	Body	0.007 [-0.036 – 0.050]	0.91		
	cg21148642	chr19	49063822	Body	0.017 [-0.032 – 0.066]	0.75		
	cg24813180	chr19	49065229	Body	-0.039 [-0.078 – 0.001]	0.20		
	cg02712700	chr19	49066585	Body	-0.142 [-0.232 – -0.052]	0.053		
	cg20896293	chr19	49068391	Body	0.042 [-0.001 – 0.086]	0.20		
	cg08900191	chr19	49071818	Body	0.145 [0.036 – 0.255]	0.10		
	cg14328676	chr19	49077983	Promoter/Body	0.153 [0.028 – 0.278]	0.12		19:49077938-49078240

cg21196201	chr19	49078045	Promoter/Body	0.100 [0.027 – 0.174]	0.095			19:49077938-49078240
cg10262032	chr19	49078119	Promoter/Body	0.044 [0.000 – 0.088]	0.20			19:49077938-49078240
cg00756734	chr19	49079029	5'UTR/1stExon/Body	-0.005 [-0.056 – 0.045]	0.95			
cg25118265	chr19	49085827	Body	-0.014 [-0.063 – 0.035]	0.81			
cg20709456	chr19	49087605	Body	-0.010 [-0.043 – 0.023]	0.79			19:49087473-49088624
cg22603982	chr19	49092366	Body	0.006 [-0.033 – 0.046]	0.92	high-CpG:53784144-53784190	chr19:49092091-49092091	19:49092049-49092599
cg22868034	chr19	49092375	Body	0.051 [0.012 – 0.089]	0.10	high-CpG:53784144-53784190	chr19:49092091-49092091	19:49092049-49092599
cg25298878	chr19	49094491	Body	-0.030 [-0.083 – 0.022]	0.53			
cg24878170	chr19	49100392	Body	0.004 [-0.065 – 0.073]	0.97			19:49100286-49100701
cg26360664	chr19	49102586	Body	0.051 [0.002 – 0.099]	0.18			

[a] Base pair position of the CpG site within the genome (human genome 19).
[b] A CpG site can be located within multiple genes or splice variants and thus can be situated within multiple gene sections.
[c] Promoter is defined as 0 to 1500 base pairs upstream of the transcription start site.
[d] Log fold change in M-values between former PICU patients and healthy controls adjusted for risk factors with corresponding 95% confidence interval.
[e] P-values extracted from multivariable linear regression models built using the limma framework, adjusted for baseline risk factors and technical variation. All P-values come from separate models.
[f] Classifications from the Functional Annotation of the Mammalian Genome (FANTOM) consortium as a low- or high-CpG density region associated with FANTOM4 promoters.
[g] Chromosomal map coordinates from the FANTOM consortium of enhancer regions associated with FANTOM5 promoters.
[h] Chromosomal map coordinates of the regulatory feature (informatically determined by the ENCODE Consortium).
Abbreviations: PICU: paediatric intensive care unit, UTR: untranslated region.

Table A3: Differentially methylated regions in former PICU patients versus healthy children

Location [a]						Difference between former patients and healthy children	
Gene name	Chromosome	Start	End	Width [b]	# CpG sites in DMR	Maximum difference [c]	Mean difference [d]
HSD17B2	chr16	82068284	82069450	1167	8	-0.018	-0.009
HSD17B2	chr16	82096585	82096624	40	3	-0.029	-0.021
HSD17B8	chr6	33173119	33173333	215	5	0.018	0.008
HSD17B10	chrX	53461213	53461462	250	7	0.017	0.009

[a] Chromosomal map coordinates of the DMR within human genome 19. DMRs were identified with the DMRcate package (Methods A2).
[b] Width in number of base pairs between first and last CpG site within the DMR.
[c] Maximum difference (expressed in M-values) between former patients and healthy children across all the CpG sites that are located within the DMR.
[d] Mean difference (expressed in M-values) between former patients and healthy children across all the CpG sites that are located within the DMR.
Abbreviations: DMR: Differentially methylated region; PICU: paediatric intensive care unit.

Table A4: Interaction of differential methylation in former PICU patients versus healthy children with sex

Gene	CpG	Chromosome	Position [a]	Gene section [b,c]	Log fold change [d]	P [e]	FANTOM4 Enhancer location [f]	FANTOM5 Enhancer location [g]	ENCODE Regulatory feature location [h]
CYP11A1	cg23808031	chr15	74659841	Promoter/1stExon	0.067 [-0.134 – 0.267]	0.51			
POR	cg10738873	chr7	75579540	5'UTR	-0.022 [-0.134 – 0.089]	0.69			
	cg17115737	chr7	75580813	5'UTR	0.040 [-0.074 – 0.154]	0.49			
	cg27372063	chr7	75601901	Body	-0.106 [-0.305 – 0.092]	0.29			7:75601477-75602026
CYB5A	cg18274065	chr18	71959833	Promoter	-0.060 [-0.180 – 0.060]	0.32			18:71958299-71959985
HSD17B1	cg02363277	chr17	40706718	Body	0.006 [-0.040 – 0.051]	0.80	high-CpG:37960203-37960365		17:40705808-40707211
HSD17B2	cg09894383	chr16	82067445	Promoter	-0.045 [-0.132 – 0.041]	0.30			
	cg11515282	chr16	82068797	Promoter	0.014 [-0.077 – 0.106]	0.76	low-CpG:80626229-80626503		
	cg20373326	chr16	82068803	Promoter	0.010 [-0.094 – 0.115]	0.84	low-CpG:80626229-80626503		
	cg19807685	chr16	82068980	5'UTR/1stExon	0.041 [-0.070 – 0.152]	0.47	low-CpG:80626229-80626503		
	cg26315602	chr16	82069046	1stExon	0.012 [-0.064 – 0.087]	0.76			
	cg00365986	chr16	82096585	Body	0.000 [-0.147 – 0.148]	0.99			
	cg05315365	chr16	82096597	Body	0.051 [-0.117 – 0.220]	0.55			
	cg13740036	chr16	82096624	Body	-0.067 [-0.246 – 0.113]	0.46			
HSD17B3	cg18014207	chr9	99015410	Body	0.009 [-0.130 – 0.149]	0.89			
HSD17B6	cg21922731	chr12	57157130	5'UTR/1stExon	-0.019 [-0.067 – 0.029]	0.44			12:57156898-57157514
HSD17B10	cg26323797	chrX	53461386	Promoter	-0.059 [-0.220 – 0.102]	0.47			X:53461138-53461763
HSD17B12	cg14262884	chr11	43757533	Body	-0.043 [-0.139 – 0.053]	0.38			
	cg21077321	chr11	43780652	Body	-0.013 [-0.163 – 0.137]	0.86			
CYP19A1	cg12009872	chr15	51520739	Body	0.003 [-0.064 – 0.070]	0.93	low-CpG:49307849-49308066		15:51520482-51520925
	cg14424631	chr15	51526420	Body	0.008 [-0.078 – 0.094]	0.85			
CYP21A2	cg11675917	chr6	31973162	Promoter	0.136 [0.004 – 0.269]	0.043			
CYP11B2	cg14389499	chr8	144000084	Promoter	0.008 [-0.104 – 0.121]	0.88			

[a] Base pair position of the CpG site within the genome (human genome 19).
[b] A CpG site can be located within multiple genes or splice variants and thus can be situated within multiple gene sections.
[c] Promoter is defined as 0 to 1500 base pairs upstream of the transcription start site.
[d] Log fold change in M-values between former PICU patients and healthy controls adjusted for risk factors with corresponding 95% confidence interval.
[e] P-values extracted from multivariable linear regression models built using the limma framework, adjusted for baseline risk factors and technical variation. All P-values come from separate models.
[f] Classifications from the Functional Annotation of the Mammalian Genome (FANTOM) consortium as a low- or high-CpG density region associated with FANTOM4 promoters.
[g] Chromosomal map coordinates from the FANTOM consortium of enhancer regions associated with FANTOM5 promoters.
[h] Chromosomal map coordinates of the regulatory feature (informatically determined by the ENCODE Consortium).
Abbreviations: PICU: paediatric intensive care unit, UTR: untranslated region.

Table A5: Interaction of differential methylation in former PICU patients versus healthy children with age at exposure

Gene	CpG	Chromosome	Position [a]	Gene section [b,c]	Log fold change [d]	P [e]	FANTOM4 Enhancer location [f]	FANTOM5 Enhancer location [g]	ENCODE Regulatory feature location [h]
CYP11A1	cg23808031	chr15	74659841	Promoter/1stExon	0.002 [-0.019 – 0.024]	0.82			
POR	cg10738873	chr7	75579540	5'UTR	-0.007 [-0.019 – 0.005]	0.26			
	cg17115737	chr7	75580813	5'UTR	-0.001 [-0.013 – 0.011]	0.84			
	cg27372063	chr7	75601901	Body	-0.013 [-0.034 – 0.008]	0.24			7:75601477-75602026
CYB5A	cg18274065	chr18	71959833	Promoter	-0.002 [-0.014 – 0.011]	0.79			18:71958299-71959985
HSD17B1	cg02363277	chr17	40706718	Body	0.001 [-0.004 – 0.006]	0.61	high-CpG:37960203-37960365		17:40705808-40707211
HSD17B2	cg09894383	chr16	82067445	Promoter	-0.012 [-0.021 – -0.002]	0.012			
	cg11515282	chr16	82068797	Promoter	-0.011 [-0.020 – -0.001]	0.029	low-CpG:80626229-80626503		
	cg20373326	chr16	82068803	Promoter	-0.005 [-0.016 – 0.006]	0.36	low-CpG:80626229-80626503		
	cg19807685	chr16	82068980	5'UTR/1stExon	-0.008 [-0.020 – 0.004]	0.18	low-CpG:80626229-80626503		
	cg26315602	chr16	82069046	1stExon	0.000 [-0.008 – 0.008]	0.93			
	cg00365986	chr16	82096585	Body	-0.009 [-0.025 – 0.007]	0.25			
	cg05315365	chr16	82096597	Body	-0.022 [-0.039 – -0.004]	0.017			
	cg13740036	chr16	82096624	Body	-0.019 [-0.039 – 0.000]	0.045			
HSD17B3	cg18014207	chr9	99015410	Body	-0.011 [-0.026 – 0.004]	0.13			
HSD17B6	cg21922731	chr12	57157130	5'UTR/1stExon	0.000 [-0.005 – 0.006]	0.87			12:57156898-57157514
HSD17B10	cg26323797	chrX	53461386	Promoter	-0.009 [-0.026 – 0.009]	0.32			X:53461138-53461763
HSD17B12	cg14262884	chr11	43757533	Body	-0.010 [-0.020 – 0.000]	0.048			
	cg21077321	chr11	43780652	Body	-0.014 [-0.030 – 0.002]	0.081			
CYP19A1	cg12009872	chr15	51520739	Body	-0.009 [-0.016 – -0.002]	0.012	low-CpG:49307849-49308066		15:51520482-51520925
	cg14424631	chr15	51526420	Body	-0.010 [-0.019 – -0.001]	0.035			
CYP21A2	cg11675917	chr6	31973162	Promoter	-0.008 [-0.022 – 0.006]	0.24			
CYP11B2	cg14389499	chr8	144000084	Promoter	0.005 [-0.007 – 0.017]	0.41			

[a] Base pair position of the CpG site within the genome (human genome 19).
[b] A CpG site can be located within multiple genes or splice variants and thus can be situated within multiple gene sections.
[c] Promoter is defined as 0 to 1500 base pairs upstream of the transcription start site.
[d] Log fold change in M-values between former PICU patients and healthy controls adjusted for risk factors with corresponding 95% confidence interval.
[e] P-values extracted from multivariable linear regression models built using the limma framework, adjusted for baseline risk factors and technical variation. All P-values come from separate models.
[f] Classifications from the Functional Annotation of the Mammalian Genome (FANTOM) consortium as a low- or high-CpG density region associated with FANTOM4 promoters.
[g] Chromosomal map coordinates from the FANTOM consortium of enhancer regions associated with FANTOM5 promoters.
[h] Chromosomal map coordinates of the regulatory feature (informatically determined by the ENCODE Consortium).
Abbreviations: PICU: paediatric intensive care unit, UTR: untranslated region.

Table A6: Differentially methylated CpG sites between former PICU patients who received glucocorticoids during their stay in the PICU versus those who did not

Gene	CpG	Chromosome	Position [a]	Gene section [b,c]	Log fold change [d]	P [e]	FANTOM4 Enhancer location [f]	FANTOM5 Enhancer location [g]	ENCODE Regulatory feature location [h]
CYP11A1	cg23808031	chr15	74659841	Promoter/1stExon	-0.189 [-0.331 – -0.047]	0.0090			
POR	cg10738873	chr7	75579540	5'UTR	0.05 [-0.031 – 0.132]	0.22			
	cg17115737	chr7	75580813	5'UTR	0.008 [-0.074 – 0.09]	0.84			
	cg27372063	chr7	75601901	Body	0.13 [-0.01 – 0.27]	0.068			7:75601477-75602026
CYB5A	cg18274065	chr18	71959833	Promoter	0.059 [-0.028 – 0.146]	0.18			18:71958299-71959985
HSD17B1	cg02363277	chr17	40706718	Body	0.016 [-0.017 – 0.048]	0.34	high-CpG:37960203-37960365		17:40705808-40707211
HSD17B2	cg09894383	chr16	82067445	Promoter	-0.001 [-0.066 – 0.064]	0.98			
	cg11515282	chr16	82068797	Promoter	-0.022 [-0.089 – 0.045]	0.52	low-CpG:80626229-80626503		
	cg20373326	chr16	82068803	Promoter	-0.036 [-0.112 – 0.039]	0.34	low-CpG:80626229-80626503		
	cg19807685	chr16	82068980	5'UTR/1stExon	0.008 [-0.073 – 0.089]	0.84	low-CpG:80626229-80626503		
	cg26315602	chr16	82069046	1stExon	-0.011 [-0.063 – 0.04]	0.66			
	cg00365986	chr16	82096585	Body	-0.110 [-0.217 – -0.002]	0.045			
	cg05315365	chr16	82096597	Body	-0.072 [-0.192 – 0.047]	0.23			
	cg13740036	chr16	82096624	Body	-0.026 [-0.155 – 0.102]	0.68			
HSD17B3	cg18014207	chr9	99015410	Body	0.101 [-0.003 – 0.205]	0.056			
HSD17B6	cg21922731	chr12	57157130	5'UTR/1stExon	0.007 [-0.028 – 0.042]	0.68			12:57156898-57157514
HSD17B10	cg26323797	chrX	53461386	Promoter	-0.076 [-0.188 – 0.036]	0.18			X:53461138-53461763
HSD17B12	cg14262884	chr11	43757533	Body	0.016 [-0.055 – 0.086]	0.66			
	cg21077321	chr11	43780652	Body	0.022 [-0.086 – 0.131]	0.68			
CYP19A1	cg12009872	chr15	51520739	Body	0.011 [-0.039 – 0.06]	0.67	low-CpG:49307849-49308066		15:51520482-51520925
	cg14424631	chr15	51526420	Body	0.095 [0.033 – 0.157]	0.0027			
CYP21A2	cg11675917	chr6	31973162	Promoter	-0.037 [-0.131 – 0.058]	0.44			
CYP11B2	cg14389499	chr8	144000084	Promoter	0.059 [-0.02 – 0.139]	0.14			

[a] Base pair position of the CpG site within the genome (human genome 19).
[b] A CpG site can be located within multiple genes or splice variants and thus can be situated within multiple gene sections.
[c] Promoter is defined as 0 to 1500 base pairs upstream of the transcription start site.
[d] Log fold change in M-values between former PICU patients and healthy controls adjusted for risk factors with corresponding 95% confidence interval.
[e] P-values extracted from multivariable linear regression models built using the limma framework, adjusted for baseline risk factors and technical variation. All P-values come from separate models.
[f] Classifications from the Functional Annotation of the Mammalian Genome (FANTOM) consortium as a low- or high-CpG density region associated with FANTOM4 promoters.
[g] Chromosomal map coordinates from the FANTOM consortium of enhancer regions associated with FANTOM5 promoters.
[h] Chromosomal map coordinates of the regulatory feature (informatically determined by the ENCODE Consortium).
Abbreviations: PICU: paediatric intensive care unit, UTR: untranslated region.

Figure A1: Speculative interpretation of potential impact of abnormal DNA methylation within steroidogenic genes on corresponding gene expression

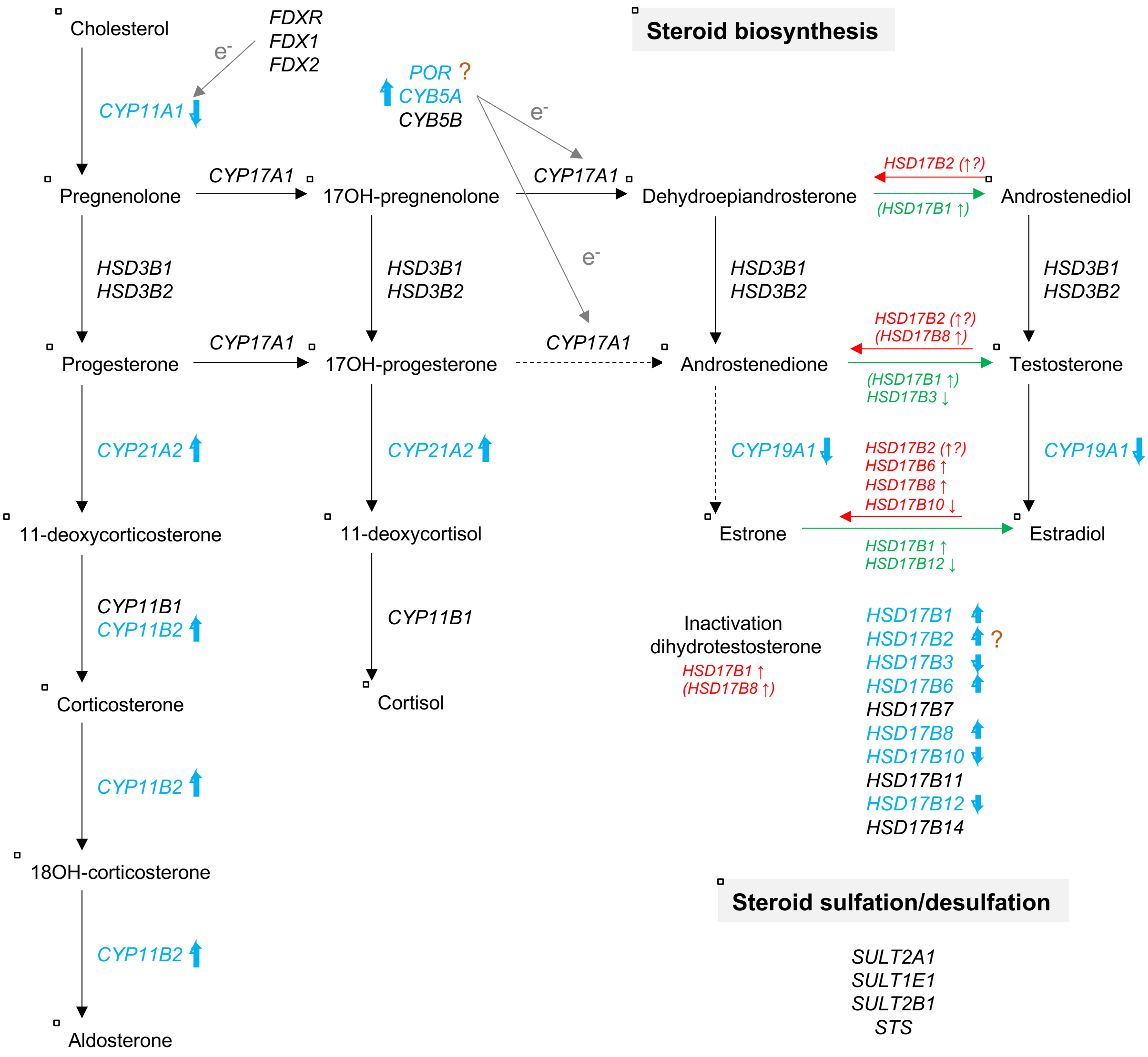

Genes that showed abnormal DNA methylation in former PICU patients as compared with healthy controls are indicated in blue. Arrows indicate assumed effects on corresponding gene expression, based on a rule of thumb that promoter/1st exon methylation mostly correlates inversely with gene expression, or alternatively based on a less uniform direct correlation between methylation of the gene body and gene expression [A2-A4]. Question marks indicate where putative gene expression effects remain ambiguous when applying these rules. 17β-hydroxysteroid dehydrogenases have different substrate specificity. Direction of the reaction catalysed by a certain enzyme is color-coded in red or green. When a gene name is placed between brackets, this corresponds to a less preferred substrate for the encoded enzyme. We emphasise that this figure only shows a speculative attempt for interpretation in the absence of corresponding gene expression data, thus not claiming ground truth. If true, however, total synthesis of steroid hormones may be downregulated, but possibly with a relative shift towards mineralo- and glucocorticoids and less production of more active sex steroid hormones.

References

A1. Peters TJ, Buckley MJ, Statham AL, Pidsley R, Samaras K, Lord RV, et al. De novo identification of differentially methylated regions in the human genome. Epigenetics & Chromatin. 2015;8:16.

A2. Jones PA. Functions of DNA methylation: islands, start sites, gene bodies and beyond. Nat Rev Genet. 2012;13:484-92.

A3. Moore LD, Le T, Fan G. DNA methylation and its basic function. Neuropsychopharmacology. 2013;38:23-8.

A4. Yang X, Han H, De Carvalho DD, Lay FD, Jones PA, Liang G. Gene body methylation can alter gene expression and is a therapeutic target in cancer. Cancer Cell. 2014;26:577-90.
